# Use of shotgun immunoproteomics for the development of protein vaccines against *Edwardsiella piscicida*

**DOI:** 10.1016/j.fsi.2025.110992

**Published:** 2025-11-08

**Authors:** Kim L. Jacobsen, Matt J. Griffin, Brett S. Phinney, Michelle Salemi, Zeinab Yazdi, Sujita Balami, Caitlin E. Older, Cynthia C. Ware, Fernando Y. Yamamoto, Noor-ul Huda, Tania Rodriguez-Ramos, Brian Dixon, Jie Ma, Benjamin R. LaFrentz, Esteban Soto

**Affiliations:** a Department of Medicine and Epidemiology, School of Veterinary Medicine, University of California, Davis, CA, 95616, USA; b Thad Cochran National Warmwater Aquaculture Center, Mississippi State University, Stoneville, MS, 38776, USA; c Proteomics Core Facility, University of California, Davis, CA, 95616, USA; d Warmwater Aquaculture Research Unit, Agricultural Research Service, U.S. Department of Aquaculture, Stoneville, MS, 38776, USA; e Department of Wildlife, Fisheries and Aquaculture, Mississippi State University, Mississippi State, MS, USA; f Department of Biology, University of Waterloo, Waterloo, Ontario, N2L 3G1, Canada; g Aquatic Animal Health Research Unit, USDA-ARS, Auburn, AL, 36832, USA

**Keywords:** Edwardsiellosis, Catfish, Salmonids, Aquaculture, Subunit vaccine, Recombinant vaccine

## Abstract

*Edwardsiella piscicida* is an important emerging pathogen in various cultured fish species. This study aimed to identify immunogenic *E. piscicida* proteins and evaluate these antigens as protein vaccines for use in aquaculture. Shotgun immunoproteomics using *anti*-*E. piscicida* serum from rainbow trout (*Oncorhynchus mykiss*) and channel catfish (*Ictalurus punctatus*) (♀) × blue catfish (*Ictalurus furcatus*) (♂) hybrids inoculated with formalin-killed whole-bacteria preparations identified 36 candidate immunogenic *E. piscicida* proteins. The chaparonin GroEL, the glycine 2TM zipper domain-containing protein (GlyZip), and coproporphyrinogen-III oxidase (COPIII) were used to orally (PO) and intra-coelomically (IC) immunize Chinook salmon (*Oncorhynchus tshawytscha*). Fish IC vaccinated with either GlyZip or COPIII demonstrated a slight, but non-significant, improvement in survival post-challenge with *E. piscicida* S11–285. Surprisingly, fish IC or PO vaccinated with GroEL displayed an anti-protective effect (RPS = − 184 % and RPS = − 76 %, respectively) against subsequent challenge. All IC vaccinated fish generated a strong specific antibody response against the immunizing protein, and sham vaccinated fish challenged with *E. piscicida* S11–285 generated a significantly higher specific antibody response to the GroEL and GlyZip proteins than negative control fish, suggesting that shotgun immunoproteomics was effective for detection of immunogenic bacterial proteins that can stimulate humoral immune responses in the host fish.

## 1. Introduction

Infectious diseases are one of the most important threats to global aquaculture, reducing the growth of farmed animals and causing devastating economic losses from morbidities and mortalities. Disease caused by *Edwardsiella* spp. are estimated to cause annual economic losses of over $50 million/year to the US aquaculture industry [1]. This genus of gram-negative bacteria consists of five distinct species, four of which are known to infect and cause disease in fish. *Edwardsiella ictaluri* is the etiologic agent of enteric septicemia of catfish (ESC) and is often associated with catastrophic losses of commercially cultured ictalurid catfish [2]. Piscine edwardsiellosis is caused by infection with one of the other three fish-pathogenic *Edwardsiella* species: *Edwardsiella tarda, Edwardsiella anguillarum,* and *Edwardsiella piscicida*. The disease is characterized by generalized septicemia and chronic granulomatous inflammation affecting a wide range of both marine and freshwater fish species [2,3].

In the past decade, *E. piscicida* has been increasingly associated with outbreaks of acute septicemia and mass mortality in economically important farmed fish species worldwide [1,4–6]. In North America, there have been reports of *E. piscicida* epizootics among farmed catfish (*Ictalurus* spp.), trout (*Oncorhynchus* spp.), barramundi (*Lates calcarifer*), and largemouth bass (*Micropterus nigricans*) [7–10]. *Edwardsiella piscicida* is a genetically diverse pathogen with at least 6 distinct phylogenetic lineages with experimentally demonstrated host-specific differences in pathogenicity [11]. Traditionally, management of edwardsiellosis in aquaculture has relied on the use of FDA-approved chemotherapeutics to treat outbreaks [2]. However, increasing evidence of *Edwardsiella* antibiotic resistance genes and associated conjugative plasmids, as well as a growing demand among consumers for antibiotic-free food, highlights a crucial need for alternative methods of disease control for *E. piscicida* [9,12–14].

Vaccination against several of the most economically important fish diseases, including furunculosis, vibriosis, and infectious salmon anemia virus, has historically proven efficacious in protecting against infection and significantly reducing antibiotic usage in aquaculture [15]. An experimental live-attenuated vaccine (LAV) against *E. ictaluri* is currently available in the U.S. under veterinary prescription and has been shown to be effective in preventing ESC among cultured catfish [16]. Currently, however, no commercially available vaccine or prophylactic protocols have been established for the prevention of *E. piscicida* in US aquaculture.

Protein vaccines, composed of one or more pathogen-derived protein antigens, represent a promising alternative to traditional treatment and vaccine modalities for prevention of piscine edwardsiellosis. One of the major advantages of protein vaccines is the improved safety compared to live-attenuated vaccines. Not only do protein vaccines lack the ability to reverse to a pathogenic state, but as they do not contain live pathogen, there is also no risk of pathogen shedding into the environment and spreading to potentially susceptible non-target species as can occur with LAVs [16,17]. Recently developed adjuvants, such as Montanide™ IMS 1312 VG (Seppic, France), as well as new protocols which enhance antigen uptake, such as liposomes and nanoparticles, have paved the way for further investigation into the novel use of protein vaccines for stimulation of cell-mediated immunity and long-term protection against intracellular pathogens such as *E. piscicida* [17–20]. In addition, because protein vaccines can be freeze-dried, they are much more shelf-stable than live-attenuated vaccines, which can be particularly important for vaccine implementation in regions where reliable cold-chain storage and transport is difficult [21].

One of the major barriers to developing effective protein vaccines is identification of immunogenic candidate antigens. Traditional strategies for antigen detection involve time-consuming and/or low throughput methodologies that often fail to account for native properties of the antigenic proteins [22,23]. *In silico* reverse vaccinology methods can be used to predict potential antigens using the pathogen genome to guide selection of candidate proteins, but accuracy of these results is dependent on the quality of the genome sequences and the accuracy of the bioinformatics programs used [24]. Additionally, results from these techniques remain informed predictions that require experimental validation to demonstrate immunogenicity. With this in mind, we elected to evaluate whether a shotgun immunoproteomic approach could be utilized to streamline screening of the bacterial proteome for immunogenic proteins and whether these proteins are effective at inducing protective immunity in fish against infection with *E. piscicida*. This approach, which was originally developed for the detection of antigenic B cell epitopes in *Francisella tularensis* and *Burkholderia pseudomallei*, involves the purification and characterization of MHC-bound bacterial peptides via liquid chromatography tandem mass spectrometry (LC-MS/MS) [22]. Identified peptides can then be immediately synthesized and tested for efficacy, thereby accelerating the protein vaccine development process. This workflow has demonstrated efficacy in identifying antigenic proteins from bacterial pathogens for mammalian species and thus presents a promising technology for the development of aquaculture vaccines [22,25].

## 2. Materials and methods

### 2.1. Experimental animals

Rainbow trout (*Oncorhynchus mykiss*), ~30–50 g in size, were obtained from the Mount Shasta Fish Hatchery (Siskiyou County, CA, USA) and arbitrarily divided into experimental and control tanks (n = 12 trout/tank) and maintained indoors at the Center for Aquatic Biology and Aquaculture (Davis, CA, USA) in 35 L tanks (18 ± 1 ^◦^C; ammonia: 0 ppm; nitrite: 0 ppm; nitrate: 40 ppm; pH: 8.0) with water delivered through a flow-through system at a rate of 4 L/min supplied with supplemental aeration using airstones. Channel catfish (*Ictalurus punctatus*) and hybrid catfish (channel catfish (♀) × blue catfish (*Ictalurus furcatus*) (♂) hybrids) were obtained as fry from a local producer and reared indoors at the Thad Cochran National Warmwater Aquaculture Center (Stoneville, MS, USA). Catfish, ~80–100 g in size, were arbitrarily divided into experimental and control tanks (n = 12 catfish/tank) and maintained in 80 L aquaria, each containing 22 L of groundwater (~24–26 ^◦^C; TAN: 1.6; chloride: 300; pH: 8.7; alkalinity: 376), with water delivered at 1.5 L/min through a precision flow-control restrictor. Dissolved oxygen was maintained near saturation using airstones supplied by a regenerative blower. For the protein vaccine trials, Chinook salmon (*Oncorhynchus tshawytscha*) obtained from the Nimbus Fish Hatchery (Gold River, CA, USA) were arbitrarily divided into experimental and control tanks (n = 12 salmon/tank) and maintained indoors at the Center for Aquatic Biology and Aquaculture (Davis, CA, USA) in identical conditions as the rainbow trout. All fish were fed a commercial extruded diet at 1 % body weight per day. Temperature for all tanks was monitored daily, and dissolved oxygen levels were measured weekly to ensure maintenance of adequate oxygenation.

Prior to all injections and blood collection procedures, trout were anesthetized via immersion in a bath containing 50 mg/L tricaine methanesulphonate (MS-222) buffered 1:1 with sodium bicarbonate, salmon were anesthetized via immersion in a bath containing 75 mg/L buffered MS-222, and catfish were anesthetized via immersion with 100 mg/L buffered MS-222.

### 2.2. Anti-Edwardsiella piscicida serum generation

Twelve archived isolates of *E. piscicida* collected from clinical cases of piscine edwardsiellosis in the United States were used in this study to generate whole cell bacterins for immunization of rainbow trout and catfish (Table 1). Isolates were revived from frozen stock on trypticase soy agar supplemented with 5 % sheep blood (SBA, University of California Davis, Biological Media Services, USA) and grown overnight at 28 ^◦^C. Bacterial colonies were harvested from SBA and resuspended in sterile PBS, then bacterial concentrations in all experiments were estimated spectrophotometrically (BioPhotometer Plus, Eppendorf AG) where a 0.5 McFarland standard of ~10^8^ CFU/mL corresponded to an optical density at 600 nm (OD_600_) of ~0.15 in phosphate-buffered saline (PBS). Bacteria quantity was confirmed via serial dilution and spot-plating of 10 μL in triplicate on SBA for colony enumeration. Whole cell bacterins were generated for each *E. piscicida* isolate through inactivation with 0.5 % formalin at 4 ^◦^C for 48 h with constant shaking. After incubation, samples were centrifuged at 3260×*g* for 30 min at room temperature and washed with PBS three times, centrifuging between each wash, then diluted in PBS to an OD of ~0.15. Formalin-killed bacterial suspensions were stored at 4 ^◦^C until use. Inactivation of bacteria was confirmed by inoculation of each sample on SBA. Then, for each isolate, 3 mL of the formalin-killed bacterial suspension was mixed with 7 mL of Montanide™ ISA 763 A (Seppic, France) adjuvant using an IKA Ultra-Turrax® Tube Drive Workstation (IKA Work, Inc., Wilmington, USA) at high speed for 30 s to generate the final vaccine preparations. The same method was used to generate sham vaccines containing 3 mL sterile PBS and 7 mL adjuvant for injection of negative control fish. All preparations were protected from light and stored at 4 ^◦^C.

Trout and catfish were inoculated via intra-coelomic (IC) injection of 0.1 mL of either the formalin-killed bacteria preparation in adjuvant or the PBS sham vaccine using a 1 mL luer-lock syringe with a 25-gauge 1” (25.4 mm) needle (n = 12 fish/treatment). This procedure was repeated 4 weeks later to booster all fish.

At 2 weeks post-booster, blood was collected from all fish via caudal venipuncture using a 3 mL or 1 mL luer-lock syringe with a 25-gauge 1” needle. Blood was immediately placed in a red top tube and stored at 4 ^◦^C for at least 1 h to allow blood to clot. After clotting, samples were centrifuged at 1449×*g* for 15 min and serum was collected and stored at − 20 ^◦^C. Following blood collection, fish were allowed to recover from anesthesia in their experimental tanks and were maintained for a further 2 weeks to allow for blood collection for whole blood bactericidal survival assays.

No mortalities among fish immunized with formalin-killed *E. piscicida* had evidence of *E. piscicida* infection on gross necropsy or microbial culture and were instead attributed to cohort aggression and/or power outages associated with inclement weather events.

### 2.3. Whole bacteria enzyme-linked immunosorbent assay (ELISA)

An ELISA was performed using protocols adapted from Heckman et al. [26] to determine the specific IgM response against the formalin-killed *E. piscicida* preparations. Briefly, a 0.5 McFarland standard of *E. piscicida* isolate S11–285 in carbonate-bicarbonate buffer (OD_600_ 0.15) was generated and then 100 μL of the bacterial suspension was added to each well of Immulon® 2HB Flat Bottom microtiter® (Thermo Fisher Scientific, USA) 96-well plates and plates incubated overnight at 4 ^◦^C. Following incubation, plates were washed three times with low salt wash buffer (LSWB; 0.02 M Trizma base, 0.38 M NaCl, 0.05 % (v/v) Tween-20, pH 7.2). Following the low salt wash, 250 μL of blocking buffer (5 % w/v skim milk powder in distilled water) was added to each well. Plates were then incubated at room temperature for 3 h and subsequently washed 3 × with LSWB. Serum from immunized and control fish were diluted 1:200 in LSWB containing 1 % bovine serum albumin (BSA; Sigma-Aldrich, USA). One hundred microliters of serum dilutions were added in duplicate to their respective wells and 100 μL of sterile PBS was added to blank control wells. Plates were incubated at 4 ^◦^C overnight. After incubation, plates were washed 3 × with high-salt wash buffer (HSWB; 0.02 M Trizma base, 0.5 M NaCl, 0.01 % (v/v) Tween-20, pH 7.7). For salmonid samples, mouse anti-trout IgM monoclonal antibodies [27] were diluted to 1:200 in sterile PBS and 100 μL of the dilution was added to each respective well. For catfish samples, 9E1 mouse anti-catfish IgM monoclonal antibodies [28], kindly donated by Dr. Eva Bengtén (Department of Cell & Molecular Biology, University of Mississippi Medical Center, Jackson, MS, USA), were diluted 1:500 in sterile PBS. Plates were incubated at room temperature for 2 h and then washed 3 × in HSWB. Goat anti-mouse IgG (H + L), horseradish peroxidase conjugate (Thermo Fisher Scientific) was diluted 1:3000 in LSWB with 1 % BSA and 100 μL of the dilution was added to each respective well and then plates were incubated for 2 h at room temperature. Following incubation, plates were washed 3 × using HSWB. The KPL ABTS® Peroxidase Substrate (SeraCare, USA) was warmed to room temperature and 100 μL of the substrate was added to each well, then plates were incubated for 1 h at room temperature. One hundred microliters of ABTS® Peroxidase Stop Solution (SeraCare) was added to each well and then the OD_410_ of the plates was measured and adjusted against PBS controls.

### 2.4. Whole blood bacterial survival assay

At 4 weeks post-booster, blood was collected from experimental and control fish (n = 3–5 per tank) via the caudal vasculature and immediately placed in lithium heparinized blood collection tubes to prevent clotting. Samples were maintained at room temperature with constant gentle inversion until processing. One-hundred microliters of heparinized whole blood from each fish was aliquoted in septuplicate in a 96-well plate and 100 μL of sterile PBS was aliquoted in triplicate on each plate to serve as a reagent control. Then, 0.5 McFarland standards of one representative isolate per bacterial clade (Table 1) in PBS were generated. The CFU of the initial inoculum was confirmed to ~1.5 × 10^8^ CFU/mL via spot plating on SBA and colony enumeration. Two microliters of each 0.5 McFarland standard (~10^5^ CFU) was added to respective wells, with one well of whole blood per fish inoculated with sterile PBS. Plates were then incubated for 24 h at 20 ^◦^C for trout whole blood and 28 ^◦^C for catfish whole blood with constant orbital shaking. Following incubation, each well was serially diluted in sterile PBS and spot plated on SBA, then incubated overnight at 28 ^◦^C for colony enumeration. When post-treatment samples were negative for the presence of bacteria on SBA, the limit of detection (LOD), defined here as 1 CFU at the lowest dilution, had been reached [29,30]. Samples at the LOD were considered less than or equal to the calculated LOD and were assigned a value of 1/2 LOD. Log reduction in bacterial growth was calculated using the following equation:

$$\mathrm{Log}\;\mathrm{Reduction}\;=\;\log_{10}(\mathrm{Inoculum},\;\mathrm{CFU}/\mathrm{mL})\;-\;\log_{10}(\mathrm{Final},\;\mathrm{CFU}/\mathrm{mL})$$

### 2.5. Protein extraction

**Fig. 1.**
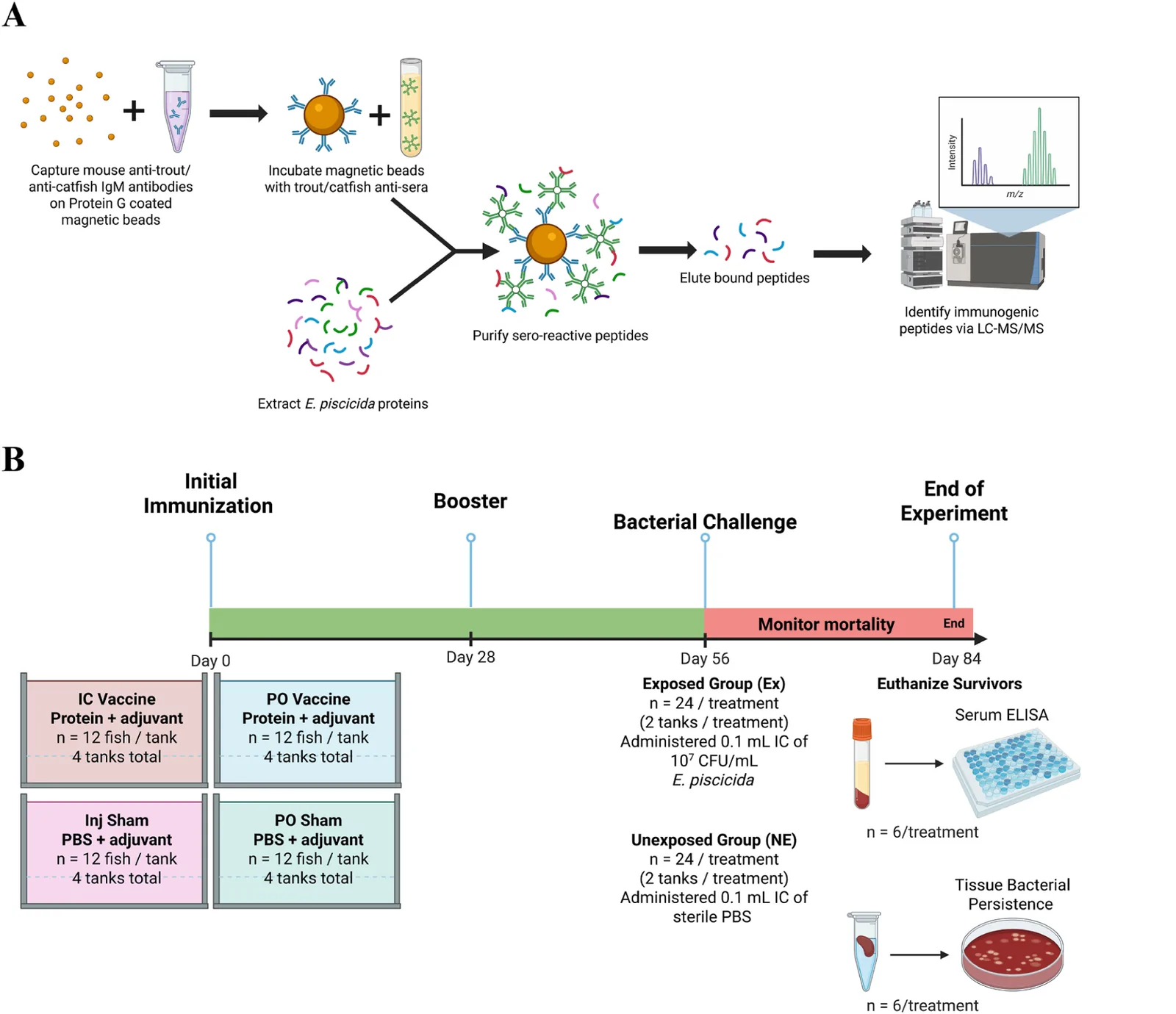
Overview of experimental design for (A) shotgun immunoproteomic identification of immunogenic *Edwardsiella piscicida* proteins and (B) *In vivo* vaccine trials. Two protein vaccine trial experiments were conducted with the first trial evaluating the GroEL protein vaccines in Chinook salmon and the second trial assessing both the COPIII and GlyZip protein vaccines in Chinook salmon. Figures generated in Biorender.

The immunoproteomic workflow utilized in this study (Fig. 1A) was adapted from protocols described by D’haeseleer et al. and Gates et al. [22,25]. *Edwardsiella piscicida* isolate S11–285 was grown to the late-exponential phase of growth in triplicate 5 mL volumes of BHI at 27 ^◦^C with constant orbital shaking. Following incubation, samples were pelleted via centrifugation at 3260×*g* for 25 min and bacterial proteins were extracted in B-PER™ reagent (Thermo Fisher Scientific) and inclusion body solubilization solution (IBS) as previously described [31].

### 2.6. Affinity purification and peptide preparation

Immunogenic proteins were purified according to manufacturer recommended protocols for the Dynabeads™ Protein G Immunoprecipitation Kit (Thermo Fisher Scientific) with specified modifications. For trout serum samples, mouse *anti*-trout IgM monoclonal antibodies were diluted 1:200 in Antibody Washing and Binding buffer and then 200 μL of the resulting dilutions were added to 50 μL aliquots of Dynabeads™ Protein G. For hybrid catfish serum samples, mouse *anti*-catfish IgM monoclonal antibodies were diluted to 1:500 in Antibody Washing and Binding buffer. Samples containing antibodies and Protein G coated magnetic beads were incubated at room temperature for 10 min with constant rotation to allow for binding of the mouse IgG antibodies to the beads. Following incubation, samples were placed on a magnet for separation of beads and the supernatant was discarded. Six pools of vaccinated serum samples for trout and for hybrid catfish were formed from pooling the serum of the five fish with the highest antibody response per vaccine group clade (V1-V6; Table 1) as determined by serum ELISA. Similar pools of serum from five negative control fish were generated from rainbow trout and hybrid catfish serum. The pooled serum samples (seven pools of rainbow trout serum and seven pools of hybrid catfish serum) were diluted 1:200 in Antibody Washing and Binding buffer and 200 μL of the resulting dilutions were mixed with the antibody-bound magnetic beads. Samples were incubated for 10 min at room temperature with constant rotation and then placed on a magnet for removal of the supernatant. Sterile PBS (100 μL) was added to each tube and then 50 μg of the B-PER™ extracted bacterial proteins and 50 μg of the IBS extracted bacterial proteins were added to each tube and gently mixed via pipetting. Tubes were incubated at room temperature for 1 h with constant rotation. After incubation, tubes were placed onto the magnet for disposal of the supernatant and the beads were washed three times with washing buffer. After washing, the beads were transferred to clean Eppendorf tubes and then three buffer exchanges using 100 μL of 50 mM tetraethylammonium bicarbonate (TEAB) and shaking for 20 min between each exchange were performed. After the last buffer exchange, trypsin was added to the TEAB in a 1:100 ratio and the solution was added to each bead sample at a volume of about 25 μL over the beads. The samples were then incubated at room temperature overnight with constant gentle shaking. After incubation, the supernatant for each sample was removed and saved in clean tubes. Beads were then washed again with enough of the 1:100 trypsin and TEAB solution to just cover the beads, then shaken for 20 min after which the supernatant was removed and saved with the previous extract.

The peptide extracts were submitted to the UC Davis Proteomics Core for digestion and protein characterization via liquid chromatography tandem mass spectrometry (LC-MS/MS).

### 2.7. Liquid chromatography tandem mass spectrometry

Peptides were resolved on a Thermo Scientific Dionex UltiMate 3000 RSLC system (Thermo Fisher Scientific) using a PepSep analytical column (PepSep, Denmark): 150 μm × 8 cm C18 column with 1.5 μm particle size (100 Å pores), preceded by a Pepmap Neo C18 5UM guard column, and heated to 40 ^◦^C. Separation was performed in a total run time of 30 min with a flow rate of 500 μL/min with mobile phases A: water/0.1 % formic acid and B: 80 % ACN/0.1 % formic acid.

### 2.8. Mass spectrometry data independent analysis

For the trout samples, peptides were directly eluted onto an Orbitrap Exploris 480 instrument (Thermo Fisher Scientific). Spray voltage was set to 1.8 kV, funnel RF level at 45, and heated capillary temperature at 275 ^◦^C. Experiments full mass spectrometry (MS) resolution was set to 60,000 at m/z 200 and full MS automatic gain control (AGC) target was 300 % with an IT of 45 msec. Mass range was set to 350–1200. AGC target value for fragment spectra was set at 1000 %. Mass Windows of 22 Da were used with an overlap of 1 Da. Resolution was set to 30,000 and IT to Auto. Normalized collision energy was set at 30 %. All data were acquired in profile mode using positive polarity, with peptide match disabled and isotope exclusion turned on.

For the catfish samples, mass spectrometry was performed on a hybrid trapped ion mobility spectrometry-quadrupole time of flight mass spectrometer (timsTOF HT, (Bruker Daltonics, Bremen, Germany) with a modified nano-electrospray ion source (CaptiveSpray, Bruker Daltonics). In the experiments described here, the mass spectrometer was operated in PASEF mode. Desolvated ions entered the vacuum region through the glass capillary and deflected into the TIMS tunnel which is electrically separated into two parts (dual TIMS). Here, the first region is operated as an ion accumulation trap that primarily stores all ions entering the mass spectrometer, while the second part performs trapped ion mobility analysis. The dual TIMS analyzer was operated at a fixed duty cycle close to 100 % using equal accumulation and ramp times of 85 ms each. Data-independent analysis (DIA) scheme consisted of one MS scan followed by MSMS scans taken with 36 precursor windows at width of 25Th per 1.09 s cycle, over the mass range 300–1200 Da. The TIMS scans layer the doubly and triply charged peptides over an ion mobility − 1/k0-range of 0.7–1.3 V*sec/cm2. The collision energy was ramped linearly as a function of the mobility from 59 eV at 1/K0 = 1.4–20 eV at 1/K0 = 0.6.

DIA data was analyzed using Spectronaut 17.5 (Biognosys Schlieren, Switzerland) using the directDIA workflow with the default settings. Briefly trypsin/P Specific was set for the enzyme allowing two missed cleavages. Fixed Modifications were set for Carbamidomethyl, and variable modification were set to Acetyl (Protein N-term) and Oxidation. For DIA search identification, PSM and Protein Group FDR was set at 0.01 %. A minimum of 2 peptides per protein group were required for quantification.

### 2.9. Protein antigen selection

Proteins were determined to be immunogenic if they had increased abundance in the affinity purified samples from immunized fish compared to negative control fish (absolute log2 ratio ≥1; *p <* 0.05). The gene ontology (GO) resource GORetriever (available at AgBase [32]) was used for obtaining biological process and molecular functional annotations of immunogenic proteins. PSORTb v3.0.3 [33] was used to predict the subcellular locations of differentially abundant proteins. Potential virulence function of candidate proteins were predicted using the “Virulence Factors of Pathogenic Bacteria Database” (VFDB; www.mgc.ac.cn/VFs/, accessed on November 1, 2023) [34]. Antigenicity of the candidate protein sequences was analyzed using the VaxiJen v2.0 online tool (http://www.ddg-pharmfac.net/vaxijen/VaxiJen/VaxiJen.html), with a prediction accuracy threshold of 0.4 (Supplemental Table 1) [35].

The coding DNA sequence for each immunogenic protein was identified from the genomes of the twelve selected *E. piscicida* isolates used to generate the formalin-killed vaccine preparations using the BLAST tool [36] in Geneious Prime v.2023.2.1 (Biomatters, CA, USA). The 12 sequences for each protein were then translated and the amino acid sequence similarity between *E. piscicida* S11–285, the isolate used for protein extraction in the shotgun immunogenic workflow, and the other 11 sequences were determined by a percent identity matrix following MAFFT alignment [37] using Geneious Prime (Supplemental Table 2).

The 36 identified immunogenic proteins were evaluated based on subcellular localization, predicted virulence function, predicted antigenicity, and high amino acid sequence conservation to select candidate protein antigens for further investigation as protein vaccines (Table 2, Supplemental Table 1, Supplemental Table 2). Candidate proteins required a predicted antigenicity of ≥0.4 and an amino acid sequence conservation of ≥99 % across all 12 *E. piscicida* isolates. Proteins meeting these requirements were additionally evaluated to select candidate proteins that had a predicted virulence function and/or were localized to the outer membrane to improve immune detection. From this analysis, three candidate proteins were selected for further investigation: Chaparonin GroEl, COPIII, and GlyZip.

### 2.10. Recombinant protein expression

The cDNA sequences for the three candidate proteins (Supplemental Table 3) were submitted to Biomatik (ON, Canada) for *Escherichia coli* expression of N-terminally His-Tagged protein. Purified protein concentrations were determined by Bradford protein assay and purity was confirmed via SDS-PAGE. Purified recombinant protein was stored at − 80 ^◦^C.

### 2.11. Protein antigen enzyme-linked immunosorbent assay (ELISA)

Indirect protein antigen ELISAs to determine the serum reactivity of immunized trout and catfish to the purified recombinant proteins were performed using similar methods as with the whole bacteria ELISAs, with specified modifications. Purified recombinant protein was diluted to 10 μg/mL in carbonate-bicarbonate buffer to serve as the coating antigen.

### 2.12. In-vivo vaccine trials

To generate protein vaccine preparations for immunization of Chinook salmon, purified recombinant protein was reconstituted in sterile PBS to a concentration of 333.3 μg/mL. For the injectable vaccine preparations, the protein suspensions were mixed 3:7 with Montanide™ ISA 763 A adjuvant using an IKA Ultra-Turrax® Tube Drive Workstation at high speed for 30 s (3 mL of 333.3 μg/mL protein suspension mixed with 7 mL of adjuvant to generate 100 μg protein/mL vaccine preparations). For the oral vaccine preparations, the protein suspensions were mixed 3:7 with Montanide™ GR1 (Seppic, France) adjuvant (3 mL of 333.3 μg/mL protein suspension mixed with 7 mL of adjuvant to generate 100 μg protein/mL vaccine preparations). Similar methods were used to generate injectable and oral sham vaccines containing PBS and adjuvant (3 mL of sterile PBS mixed with 7 mL of adjuvant). All preparations were protected from light and stored at 4 ^◦^C.

In the first vaccine trial (Fig. 1B), Chinook salmon were vaccinated either via IC injection with 0.1 mL of the vaccine preparation containing 100 μg/mL GroEL protein suspended in Montanide™ ISA 763 A (10 μg protein/fish, n = 48, quadruplicate tanks with 12 fish/tank) or oral gavage with 0.1 mL of the vaccine preparation containing 100 μg/mL GroEL protein suspended in Montanide™ GR1 (10 μg protein/fish, n = 48, quadruplicate tanks with 12 fish/tank). Sham vaccinated fish were either injected with 0. l mL of sterile PBS suspended in Montanide™ ISA 763 A (n = 48, quadruplicate tanks with 12 fish/tank) or orally gavaged with 0.1 mL of sterile PBS suspended in Montanide™ GR1 (n = 48, quadruplicate tanks with 12 fish/tank). This procedure was repeated at 4 weeks post-initial immunization to booster all fish.

At 4 weeks post-booster, half of the fish in each treatment group (n = 24 fish/treatment) were challenged via IC injection of ~10^6^ CFU/fish of *E. piscicida* isolate S11–285 suspended in PBS. The other half of the fish received IC injection of an equivalent volume of sterile PBS. Fish were monitored for 4 weeks post-challenge, during which period fish were observed twice daily, and any mortalities were recorded and sampled via aseptic swabbing of the posterior kidney and brain and inoculation on SBA for re-isolation of *E. piscicida*. After removal of mortalities, the feed amount was adjusted to prevent deterioration in water quality. At 28 days post challenge (dpc), all surviving fish were euthanized, and serum was harvested from all fish as previously described. Additionally, spleen and posterior kidney samples from three fish per tank (n = 6 per treatment) were collected for assessment of tissue bacterial persistence.

In the second vaccine trial, Chinook salmon were initially immunized via IC injection with 0.1 mL of the vaccine preparation containing 100 μg/mL COPIII protein suspended in Montanide™ ISA 763 A (10 μg protein/fish, n = 48), IC injection with 0.1 mL of 100 μg/mL GlyZip protein suspended in Montanide™ ISA 763 A (10 μg protein/fish, n = 48), oral gavage with 0.1 mL of 100 μg/mL COPIII protein suspended in Montanide™ GR1 (10 μg protein/fish, n = 48), or oral gavage with 0.1 mL of 100 μg/mL GlyZip protein suspended in Montanide™ GR1 (10 μg protein/fish, n = 48). Control fish were sham vaccinated either through IC injection with 0. l mL of sterile PBS suspended in Montanide™ ISA 763 A (n = 48) or via oral gavage with 0.1 mL of sterile PBS suspended in Montanide™ GR1. Fish were all boostered at 4 weeks post-vaccination. At 4 weeks post-booster, half of the fish in each treatment group were challenged via IC injection of ~10^6^ CFU/fish of *E. piscicida* isolate S11–285 suspended in PBS (n = 24 fish/treatment) and the other half of the fish were administered 0.1 mL sterile PBS IC. Fish were monitored and sampled in an identical manner as the first vaccine trial.

### 2.13. Cytokine sandwich enzyme-linked immunosorbent assay

Serum IL-2 levels of 28 dpc salmon were measured via sandwich ELISA following previously described protocols [38,39]. Serum concentrations were calculated from linear regressions of the standard curve using GraphPad Prism v10.0.1.

### 2.14. Western blot

SDS-PAGE and Western blot was performed following the method described by LaFrentz et al. (2004) [40]. Briefly, whole proteins were extracted from Chinook salmon head kidney tissue using light sonication and NP-40 lysis buffer supplemented with protease inhibitors (150 mM NaCL, 50 mM Tris-HCl pH 8.0, 1 % Triton X-100, and 1X Halt protease and phosphatase inhibitor cocktail). Protein concentration of the sample was determined using a Micro BCA Protein Assay Kit according to manufacturer’s directions (Thermo Fisher Scientific). A total of 28 μg of protein per well was separated via SDS-PAGE in Criterion TGX Stain-Free AnyKD gels and transferred to a 0.2 μM nitrocellulose membrane using Trans-Blot Turbo transfer packs and the Mixed MW program of the Trans-Blot Transfer System (Bio-Rad Laboratories, USA). After blocking with 4 % non-fat dry milk in PBS, Chinook salmon serum samples (control and GroEL vaccinated; n = 5 each) were applied to individual membrane strips at a 1:50 dilution in PBS-T and incubated overnight with gentle shaking at 4 ^◦^C. After subsequent washes, the membrane was treated for 1 h with mAb 1.14 (mouse anti-rainbow trout IgM with specificity to Chinook salmon IgM) at a 1:50 dilution in PBS-T at room temperature, washed, and then treated with goat anti-mouse IgG alkaline phosphatase-conjugated antibody at a 1:3000 dilution for 1 h. Membranes were washed and then immunogenic proteins were visualized by the addition of 1 × AP color development solution (Bio--Rad Laboratories) and images captured using a Bio-Rad ChemiDoc Touch Imaging System (Bio-Rad Laboratories).

### 2.15. Tissue bacterial persistence assay

Spleen and posterior kidney samples from salmon 28 dpc (n = 6/treatment) were weighed and then manually homogenized in 0.5 mL of sterile PBS. Tissue homogenates were then serially diluted in sterile PBS and 10-fold dilutions were plated in triplicate on SBA and incubated at 28 ^◦^C overnight for colony enumeration. Colony counts were normalized by tissue weight for statistical analysis.

### 2.16. Statistical analysis

Statistical analyses for ELISAs, survival data, and whole blood bacterial survival assays were performed in GraphPad Prism v10.0.1. ANOVA or Welch ANOVA tests, depending on the data distribution, followed by Dunnett’s tests were used to assess differences in specific antibody responses, measured by ELISA, of vaccinated fish and negative control fish. Tukey’s tests were used to compare host specific antibody response based on clade of the inoculating bacteria or the host fish species. Similarly, one-Way ANOVA with Dunnett’s tests were used to assess for differences in bacterial survival in whole blood of immunized fish compared to negative control. ANOVA with Dunnett’s tests were used to compare tissue bacterial load between vaccinated and sham vaccinated salmon. Data are given as the mean value ± standard error of the mean. The value of *p <* 0.05 was considered significant for all statistical analyses. Analyses of proteomic and immunoproteomic data were performed in Spectronaut® (Biognosys Inc, USA). Proteins with a *p*-value of *<*0.05, as determined by unpaired Student’s t-test, and an absolute log2 ratio ≥1 were considered differentially expressed. The protective efficacy of the vaccine formulations was assessed by calculating the cumulative percent mortality (CPM) of each treatment group and the relative percent survival (RPS) of the vaccinated groups 28 dpc [41]. Differences in post-challenge survival between vaccinated and unvaccinated fish were calculated using ANOVA with Dunnett’s tests.

## 3. Results

### 3.1. Channel catfish and hybrid catfish had higher immunoreactivity to E. piscicida S11–285 than rainbow trout

**Fig. 2.**
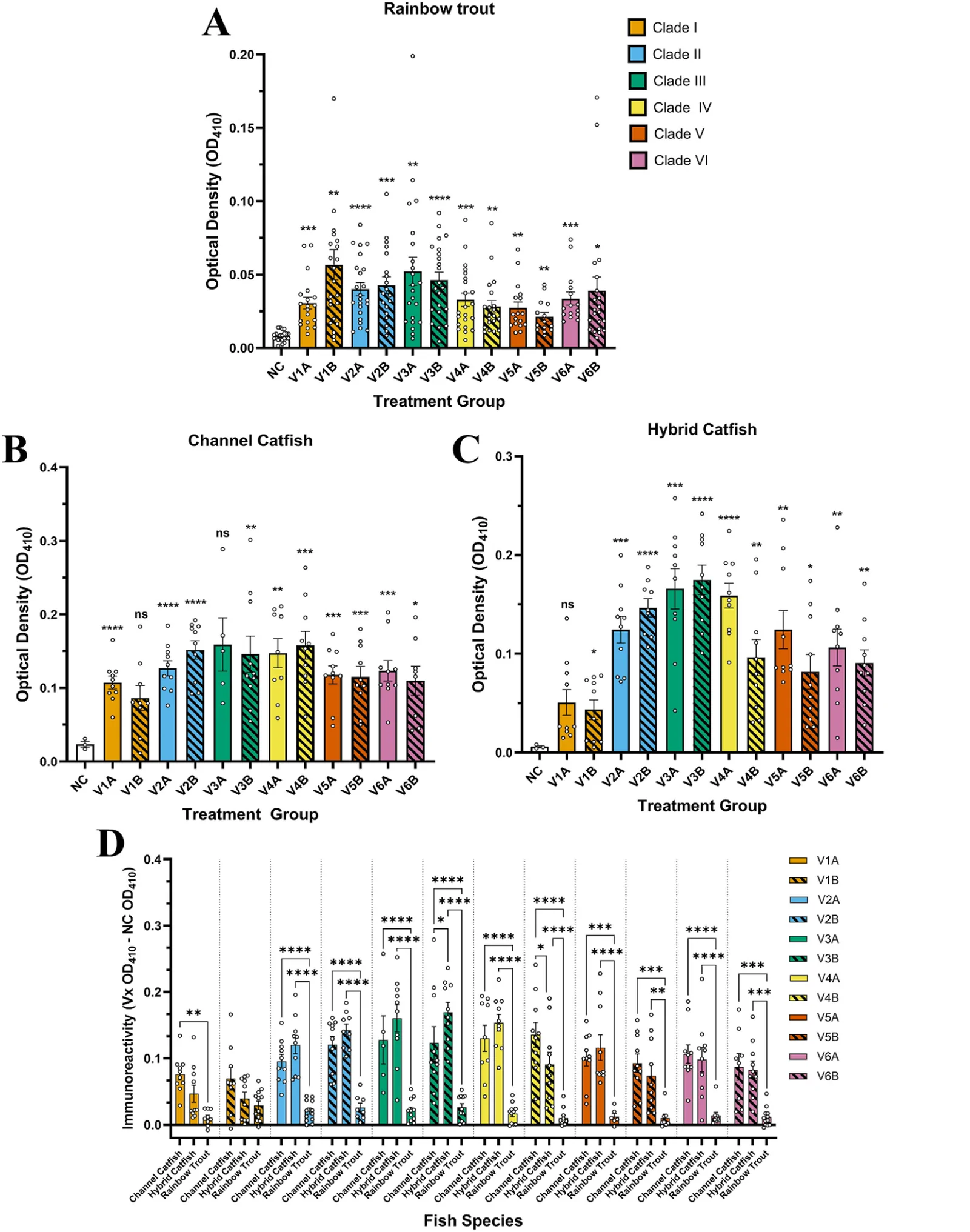
Specific *anti*-*Edwardsiella piscicida* S11–285 IgM antibody response of immunized (A) rainbow trout, (B) channel catfish, and (C) hybrid catfish as determined by indirect ELISA. Brown-Forsythe ANOVA and Welch ANOVA tests were used to assess differences in immunoreactivity among groups, with post-hoc Dunnett’s T3 tests to compare difference between individual groups and the negative control group. (D) Immunoreactivity of fish serum to *E. piscicida* isolate S11–285 was determined as the difference between the optical densities (OD) of immunized fish serum to the mean OD of the corresponding negative control serum group; differences in immunoreactivity were determined by Brown-Forsythe ANOVA and Welch ANOVA tests with post-hoc Tukey’s multiple comparisons tests. Color reflects isolate bacterial clade for formalin-killed bacterial preparations: Clade I: orange; Clade II: blue; Clade III: green; Clade IV: yellow; Clade V: red; Clade VI: pink. Error bars represent standard error (SEM). For A-C, asterisks indicate statistical significance between immunized groups and negative control as determined by Dunnett’s T3 multiple comparisons tests. For D, asterisks indicate statistical significance between fish species as determined by Tukey’s multiple comparisons tests. ns: not significant, *p >* 0.05; *: *p <* 0.05; **: *p <* 0.01; ***: *p <* 0.001; ****: *p <* 0.0001. NC, negative control. (For interpretation of the references to color in this figure legend, the reader is referred to the Web version of this article.)

Immunization with the formalin-killed *E. piscicida* + Montanide™ ISA 763 A preparations stimulated a significant humoral response against *E. piscicida* in the rainbow trout, channel catfish, and hybrid catfish (Fig. 2). Serum IgM levels against *E. piscicida* isolate S11–285 were significantly higher (*p <* 0.05) in almost all vaccine groups for all three fish species as compared to negative control fish. All immunized rainbow trout had significantly higher IgM responses against *E. piscicida* S11–285 compared to control trout (Fig. 2A). All immunized hybrid catfish except for those vaccinated with V1A (Fig. 2C) and all immunized channel catfish except for those vaccinated with V1B or V3A (Fig. 2B) had significantly higher IgM responses against *E. piscicida* S11–285 compared to control catfish.

For almost all vaccine groups the levels of specific *anti*-*E. piscicida* isolate S11–285 antibodies in rainbow trout serum were significantly lower than the levels of specific antibody present in channel catfish serum (Fig. 2D). Fish vaccinated with V1B were the only exception in which there was no significant species-associated difference in specific antibody levels. Additionally, for all vaccine groups except for those derived from Clade I isolates (V1A, V1B) the levels of specific antibody present in rainbow trout serum was significantly lower than the levels in hybrid catfish serum. Hybrid catfish immunized with formalin-killed *E. piscicida* 35–70 (V3B) had significantly higher specific antibodies against *E. piscicida* S11–285 than channel catfish immunized similarly, while conversely channel catfish immunized with formalin-killed *E. piscicida* S12–281 (V4B) had significantly higher specific antibody levels than hybrid catfish immunized similarly. There were no significant differences in the levels of specific antibodies between hybrid and channel catfish for any of the other vaccine groups.

No mortalities among fish immunized with formalin-killed *E. piscicida* had evidence of *E. piscicida* infection on gross necropsy or microbial culture and were instead attributed to cohort aggression and/or power outages associated with inclement weather events.

### 3.2. Bacterial survival was reduced in immunized rainbow trout whole blood but not catfish blood

**Fig. 3.**
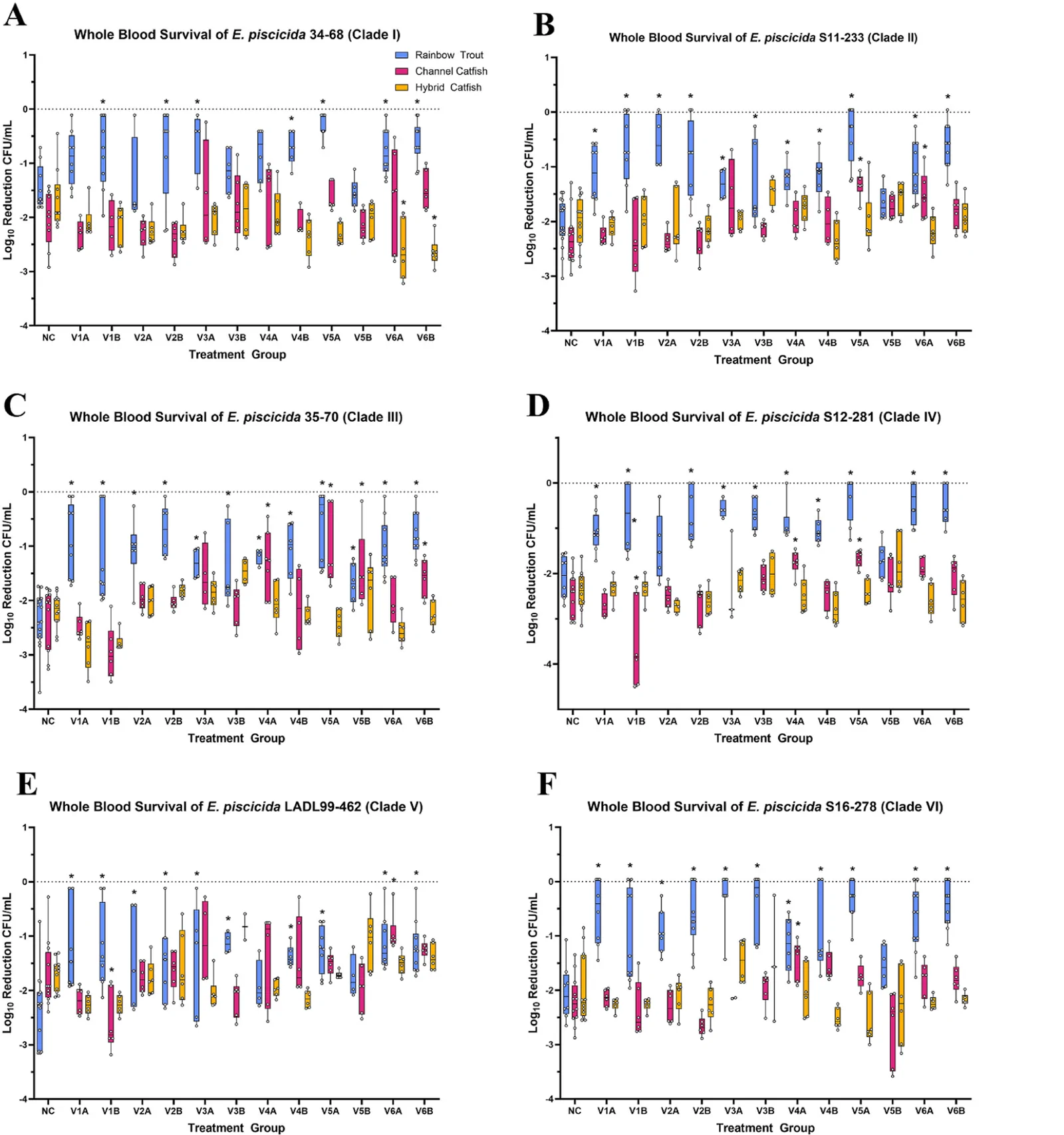
Log reduction of *Edwardsiella piscicida* isolates (A) 34–68, Clade I; (B) S11–233, Clade II; (C) 35–70, Clade III; (D) S12–281, Clade IV; (E) LADL99–462, Clade V; and (F) S16–278, Clade VI after incubation for 24 h in whole heparinized blood from rainbow trout, channel catfish, and hybrid catfish. The dotted line indicates the limit of detection for bacterial enumeration. Asterisks indicate statistical significance (*p <* 0.05) from the corresponding negative control whole blood group for each fish species as determined by Dunnett’s multiple comparisons tests.

Selected *E. piscicida* isolates from each of the six bacterial clades (Table 1) had reduced 24-h survival in whole blood from rainbow trout immunized with formalin-killed bacterial preparations compared to whole blood from negative control trout exposed to the sham vaccine (Fig. 3). *Edwardsiella piscicida* isolate 35–70 (Clade III; Fig. 3C) had significantly higher log reduction (reduced bacterial survival; *p <* 0.05) of bacteria post 24-h incubation in rainbow trout whole blood from all vaccine groups compared to negative control trout. *Edwardsiella piscicida* isolates S11–233 (Clade II; Fig. 3B) and S16–278 (Clade VI; Fig. 3F) had significantly reduced survival in rainbow trout whole blood from all vaccine groups compared to negative control except for whole blood from trout immunized with V5B, for which there was no significant difference in log reduction of bacteria compared to negative control. *Edwardsiella piscicida* isolate S12–281 (Clade IV; Fig. 3D) had significantly reduced survival of bacteria in rainbow trout whole blood from all vaccine groups except for whole blood from trout immunized with V2A and V5B. For *E. piscicida* isolate LADL99–462 (Clade V; Fig. 3E), there was significantly higher log reduction of bacterial survival in rainbow trout whole blood from all vaccine groups except for whole blood from trout immunized with V4A and V5B. *Edwardsiella piscicida* isolate 34–68 (Clade I; Fig. 3A) had the greatest survival in immunized rainbow trout whole blood of the six isolates tested, with no significant difference in bacterial survival in whole blood from rainbow trout immunized with V2A, V3B, V4A, and V5B.

Immunization with formalin-killed *E. piscicida* did not consistently reduce bacterial survival in whole blood from channel catfish or hybrid catfish. Additionally, bactericidal activity of whole blood did not consistently correlate between the bacterial clade used to inoculate the fish and the bacterial clade used in the survival assay. In some cases, bacterial survival in immunized catfish whole blood was higher than in negative control whole blood. There was no significant difference in the log reduction of *E. piscicida* isolate 34–68 (Clade I) post 24-h incubation in the whole blood of immunized channel catfish, compared to negative control channel catfish. *Edwardsiella piscicida* isolate S16–278 (Clade VI) only had significantly reduced bacterial survival in channel catfish whole blood from catfish immunized with V4A, compared to negative control catfish. *Edwardsiella piscicida* isolate S11–233 (Clade II) had significantly decreased survival of bacteria only in channel catfish whole blood from catfish immunized with V5A and V6A. For *E. piscicida* isolate 35–70 (Clade III), there was significantly reduced bacterial survival only in channel catfish whole blood from catfish immunized with V4A, V5A, V5B, and V6B. *Edwardsiella piscicida* isolate S12–281 (Clade IV) had significantly increased log reduction in channel catfish whole blood from catfish immunized with formalin-killed *E. piscicida* isolates V4A and V5A, but also had significantly decreased log reduction (improved bacterial survival; *p <* 0.05) in whole blood from channel catfish immunized with V1B. Channel catfish whole blood survival of *E. piscicida* isolate S16–278 (Clade VI) showed similarly mixed results with reduced bacterial survival in whole blood from catfish immunized with V6A, but significantly increased survival in whole blood from catfish immunized with V1B.

For hybrid catfish, there was no significant difference in 24-h log reduction for *E. piscicida* isolates S11–233, 35–70, S12–281, LADL99–462, and S16–278 when incubated in immunized hybrid catfish whole blood compared to negative control hybrid catfish whole blood. Only *E. piscicida* isolate 34–68 (Clade I) showed a significant difference in bacterial whole blood survival for hybrid catfish, with improved survival in hybrid catfish whole blood from catfish immunized with V6A and V6B.

### 3.3. Selection of immunogenic protein antigens

Antisera from rainbow trout and hybrid catfish immunized with formalin-killed *E. piscicida* was used to identify immunogenic *E. piscicida* protein antigens via LC-MS/MS (absolute log2 ratio ≥1; *p <* 0.05). Fourteen unique *E. piscicida* proteins were determined to be immunogenic in the rainbow trout and 23 proteins were immunogenic in the hybrid catfish (Table 2, Supplemental Table 1). Of these proteins, the majority were localized to the cytoplasm (13/14 for rainbow trout; 13/23 for hybrid catfish) and were involved in housekeeping functions such as translation and metabolism. The rainbow trout and hybrid catfish immunoproteomes shared only one protein, the small ribosomal subunit protein uS17. Structural constituents of ribosomes were the most abundant protein group in both immunoproteomes (8/14 for rainbow trout; 6/23 for hybrid catfish). The rainbow trout immunoproteome contained one protein with predicted localization to the periplasm, superoxide dismutase, while the hybrid catfish immunoproteome contained four proteins localized to the cytoplasmic membrane; DUF883 domain-containing protein, Sec translocon accessory complex subunit YajC, Phosphatidylserine decarboxylase proenzyme, and (Fe-S)-binding protein; and one protein localized to the outer membrane (Glycine zipper 2TM domain-containing protein). All identified immunogenic proteins had high sequence homology (pairwise identity ≥98 %) among the twelve selected *E. piscicida* isolates used to generate the formalin-killed vaccine preparations (Supplemental Table 2). Most of the identified proteins were predicted probable antigens (12/14 for rainbow trout; 18/23 for hybrid catfish) based on their Vaxigen v2.0 Value (threshold 0.4).

### 3.4. The oral and injectable GroEL vaccines induced an anti-protective effect against subsequent challenge

**Fig. 4.**
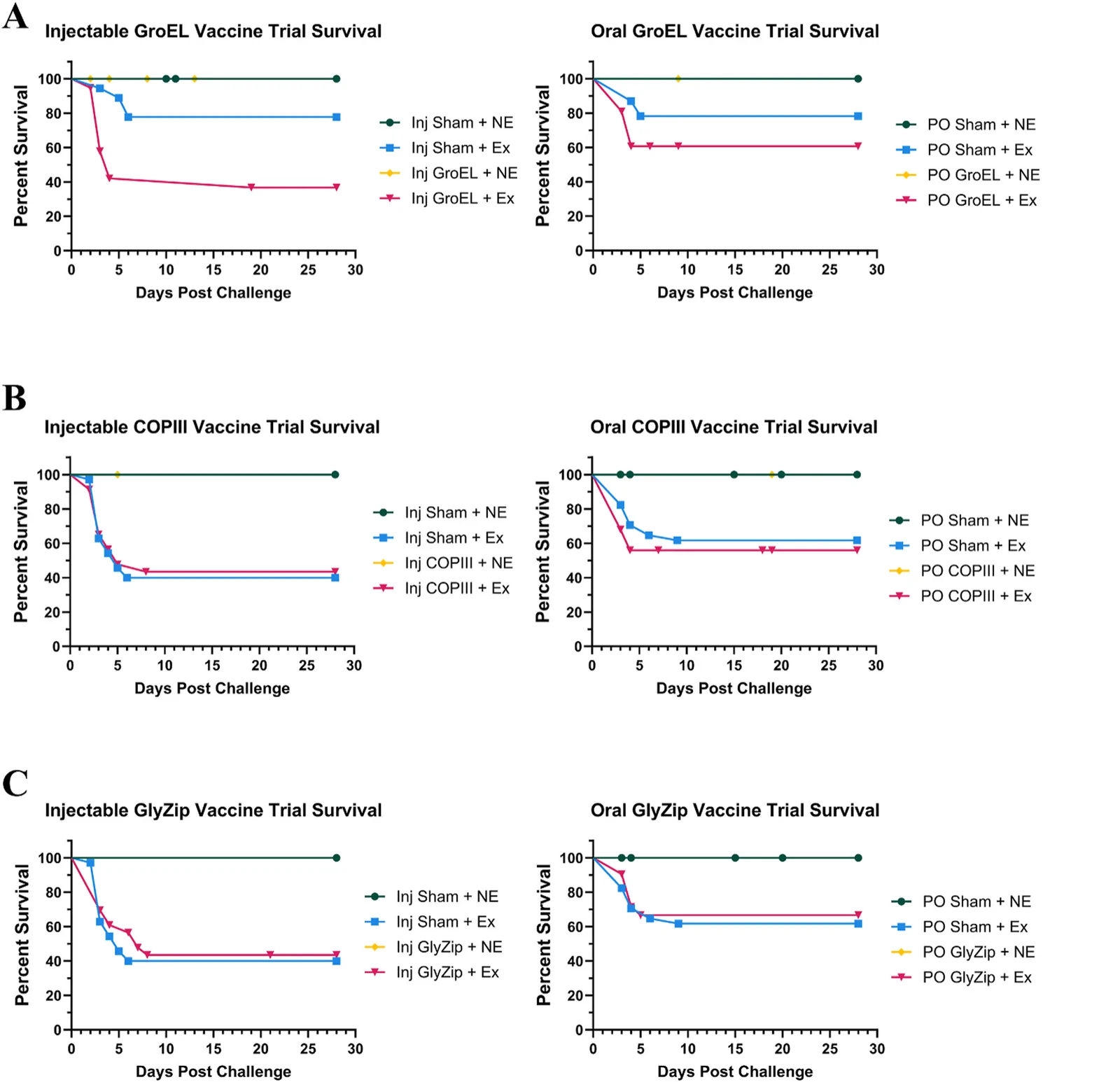
Survival of vaccinated and sham vaccinated Chinook salmon after challenge with *Edwardsiella piscicida* S11–285. Each treatment was performed in duplicate with 12 fish per tank (n = 24 fish/treatment). Inj, intra-coelomically injected; PO, orally gavaged; NE, not exposed to *E. piscicida* S11–285; Ex, challenged with *E. piscicida* S11–285.

The first vaccine trial evaluated GroEL as a protein vaccine candidate against *E. piscicida* in Chinook salmon. Mortality in fish challenged with *E. piscicida* S11–285 began on day 2 post-challenge, with peak mortality occurring on days 3–5 post-challenge (Fig. 4A). This is in line with previous challenge experiments with *E. piscicida* in Chinook salmon [11]. At 28 dpc there was no mortality in any of the unchallenged groups. The injectable and oral sham vaccinated fish challenged with *E. piscicida* S11–285 achieved a CPM of 22.2 % and 21.7 %, respectively (Table 3). Surprisingly, both the oral and injectable GroEL vaccines appeared to induce an anti-protective effect in the salmon post-challenge. Fish vaccinated with the oral GroEL preparation had an RPS of − 76 % whist those vaccinated with the injectable GroEL formulation had an RPS of − 184 %.

### 3.5. Intra-coelomic injection of COPIII and GlyZip vaccines induced a slight, but nonsignificant, improvement in post-challenge survival

In the second vaccine trial, both the COPIII and GlyZip proteins were evaluated as protein vaccine candidates in Chinook salmon. Similar to the first trial, peak mortality occurred 3–5 dpc (Fig. 4B–C). The injectable and oral sham vaccinated fish challenged with *E. piscicida* S11–285 achieved a cumulative percent mortality (CPM) of 60 % and 38.2 %, respectively. The injectable COPIII and GlyZip vaccines induced slight, but non-significant (*p >* 0.05) increases in post-challenge survival (RPS = 6 % and 9.7 %, respectively; Table 3). The COPIII oral vaccine preparation induced a non-significant decrease in post-challenge survival (RPS = − 15 %), whilst conversely the fish vaccinated with the oral GlyZip preparation showed a slight, but non-significant, increase in post-challenge survival (RPS = 13 %).

### 3.6. Salmon generated strong specific IgM responses against the candidate proteins

**Fig. 5.**
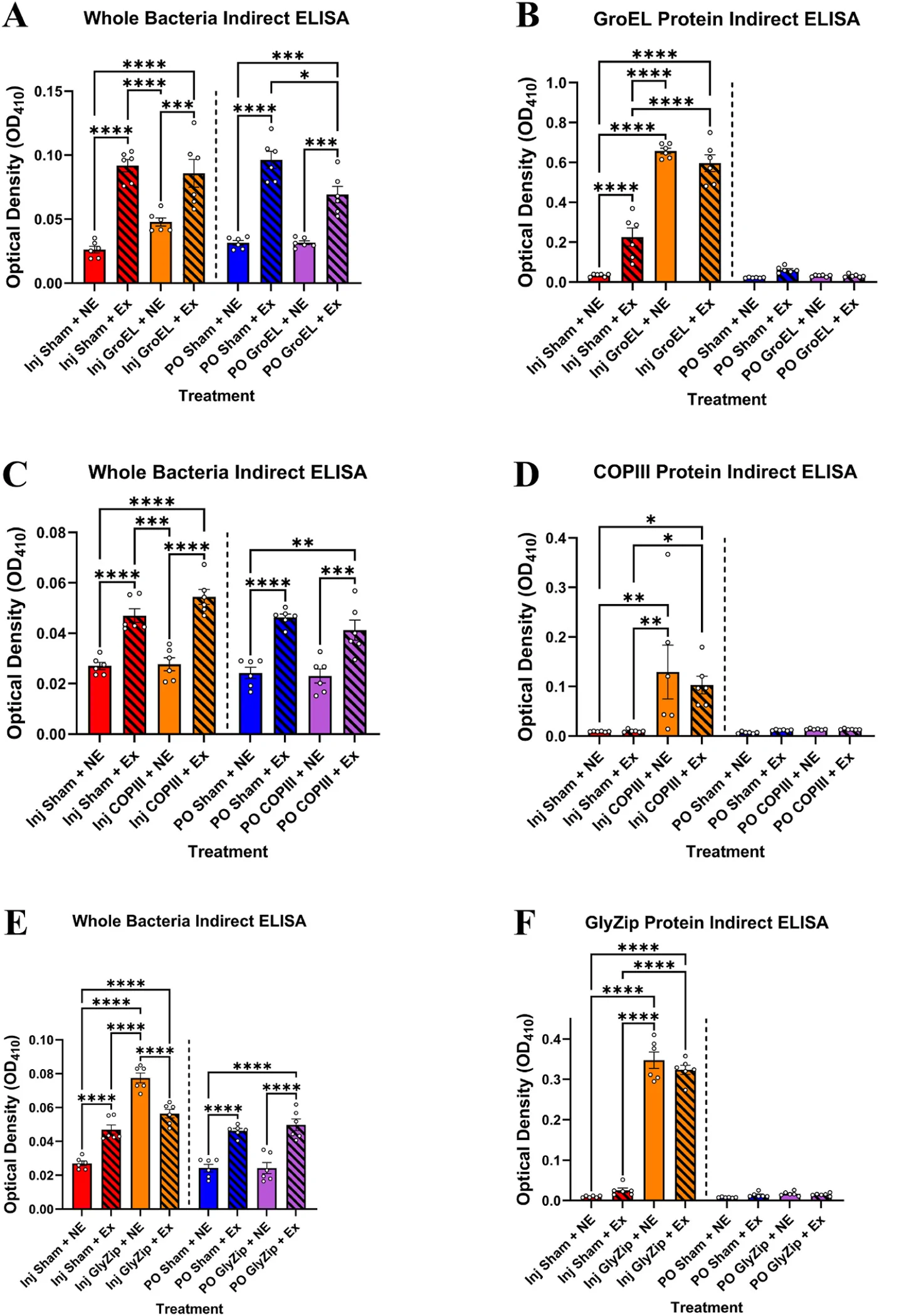
Specific antibody (IgM) levels against *Edwardsiella piscicida* S11–285 and purified recombinant protein in surviving Chinook salmon (mean ± SE, n = 6) at 28 dpc as determined by serum ELISA. Serum IgM response of GroEL vaccinated (and sham vaccinated) fish against (A) whole bacteria *E. piscicida* S12–285 or (B) purified GroEL protein. Serum IgM response of COPIII vaccinated (and sham vaccinated) fish against (C) whole bacteria *E. piscicida* S12–285 or (D) purified COPIII protein. Serum IgM response of GlyZip vaccinated (and sham vaccinated) fish against (E) whole bacteria *E. piscicida* S12–285 or (F) purified GlyZip protein. Asterisks indicate a significant difference (*p <* 0.05) from the corresponding control as determined by Tukey’s multiple comparisons tests: *: *p <* 0.05; **: *p <* 0.01; ***: *p <* 0.001; ****: *p <* 0.0001. Inj, Injectable vaccine preparation; PO, oral vaccine formulation; NE, non-exposed; Ex, exposed.

Serum from surviving salmon 28 dpc was used in indirect ELISAs to determine specific IgM antibody responses against whole bacteria *E. piscicida* S11–285 and to the purified proteins used to generate the vaccine formulations (Fig. 5). As expected, all vaccinated and sham vaccinated fish challenged with *E. piscicida* S11–285 generated a significantly increased *anti*-*E. piscicida* IgM antibody response compared to unchallenged fish. Unchallenged fish vaccinated with the injectable GlyZip vaccine preparation demonstrated an increased specific *anti*-*E. piscicida* IgM response compared to both challenged and unchallenged sham vaccinated fish (Fig. 5E). All IC vaccinated fish, regardless of exposure status, also generated an increased IgM response against the immunizing protein (Fig. 5B, D, 5F) compared to sham vaccinated fish; however, no PO vaccinated fish developed an increased antibody response against the immunizing protein. Additionally, IC sham vaccinated fish exposed to *E. piscicida* S11–285 had a significantly higher *anti*-GroEL IgM response compared to negative control fish.

### 3.7. Vaccination with selected proteins did not significantly alter long-term tissue bacterial persistence

**Fig. 6.**
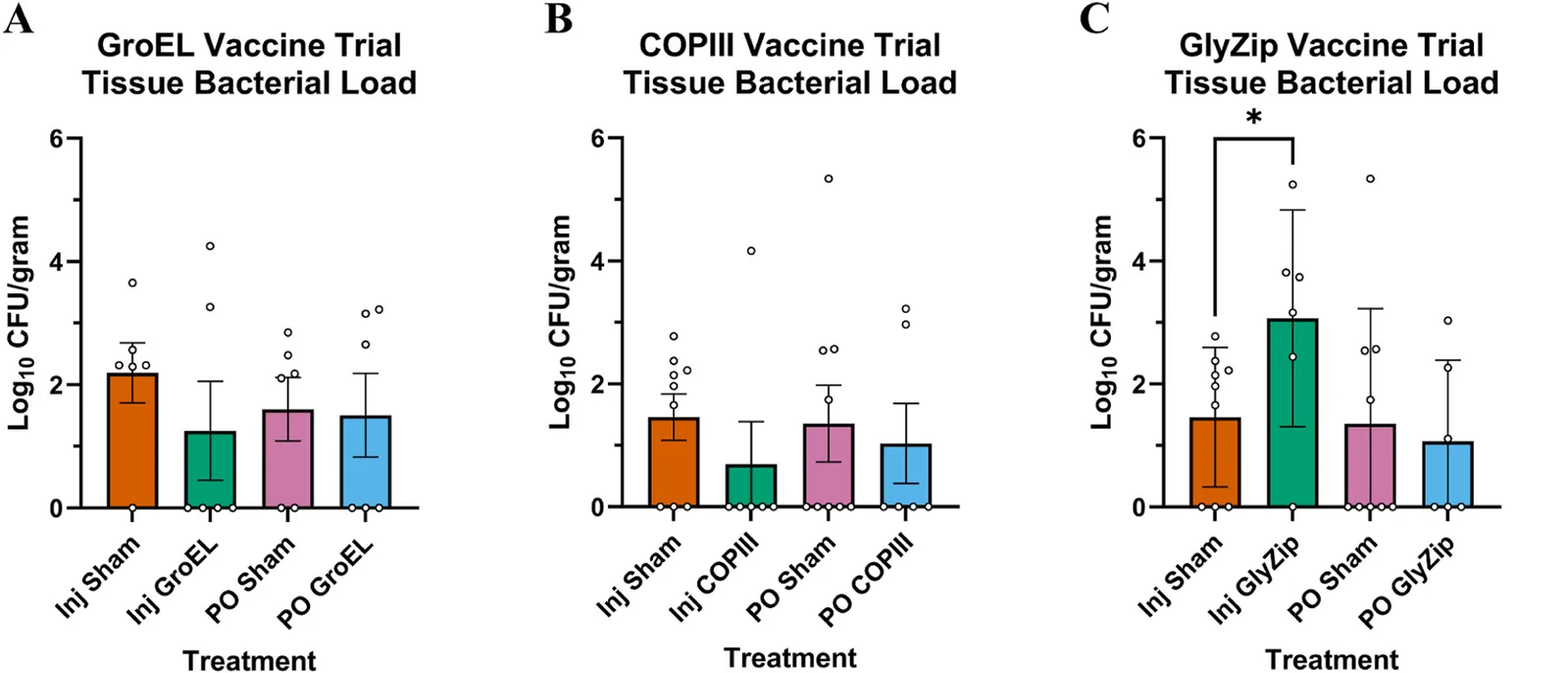
Bacterial load in pooled posterior kidney and spleen samples from vaccinated and sham vaccinated Chinook salmon 28 days post-challenge (n = 6/treatment). Colony counts after incubation overnight on SBA were normalized against the weight of the tissue and present as Log_10_ CFU/g of tissue. Asterisks indicate a significant difference (*p <* 0.05) in bacterial load between vaccinated and sham vaccinated fish exposed to *E. piscicida* S11–285 as determined by ANOVA with Dunnett’s tests. Inj, intra-coelomic injectable vaccine preparation; PO, oral vaccine preparation.

At 28 dpc, pooled spleen and posterior kidney samples from 6 fish/treatment were homogenized and plated on SBA to determine long-term persistence of *E. piscicida* in the tissues of vaccinated and unvaccinated salmon. Samples from unexposed fish did not grow *E. piscicida* colonies on SBA after overnight incubation at 28 ^◦^C (data not shown). Fish vaccinated with the injectable GlyZip vaccine preparation had significantly higher tissue bacterial load at 28 dpc compared to sham vaccinated fish (Fig. 6). There was no significant difference in tissue bacterial load between vaccinated and sham vaccinated fish for any of the other treatments.

### 3.8. Intra-coelomic vaccination did not significantly alter 28 dpc serum IL-2 levels

**Fig. 7.**
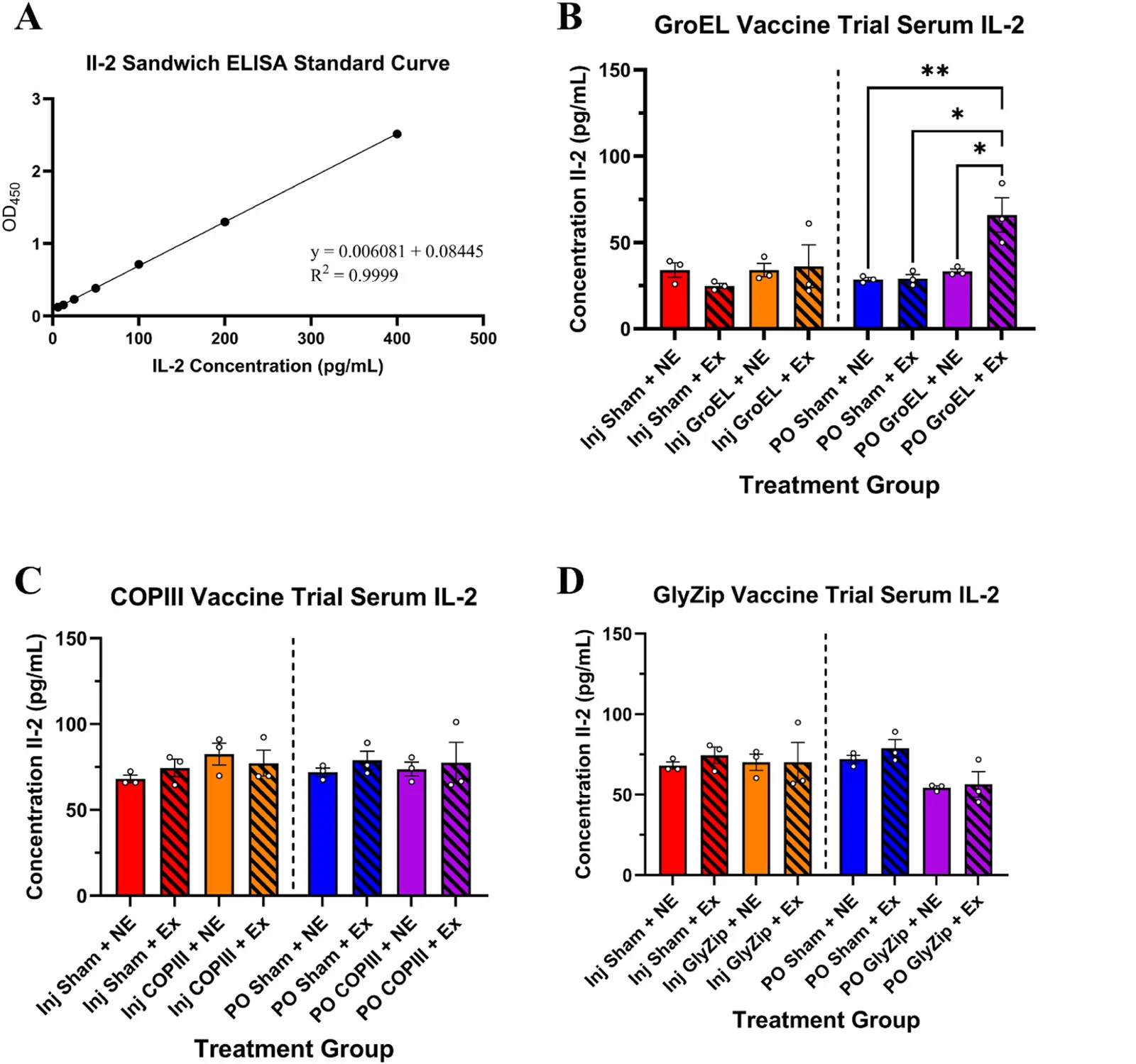
Serum Il-2 levels from Chinook salmon 28 dpc as determined by sandwich ELISA. (A) Linear standard curve of sandwich ELISA for the concentration measurement of serum IL-2 from vaccinated and unvaccinated salmon in the GroEL (B), COPIII (C), and GlyZip (D) vaccine trials. Asterisks indicate a significant difference (*p <* 0.05) from the corresponding control as determined by Dunnett’s T3 multiple comparisons tests: *: *p <* 0.05; **: *p <* 0.01. Inj, Injectable vaccine preparation; PO, oral vaccine formulation; NE, non-exposed; Ex, exposed.

Sandwich ELISA using serum from salmon 28 dpc did not reveal any significant differences in circulating IL-2 levels between vaccinated and unvaccinated or exposed and unexposed fish in the COPIII and GlyZip vaccine trial (Fig. 7). Similarly, in the GroEL vaccine trial, fish administered the injectable vaccine preparation did not have significantly different serum IL-2 levels compared to sham vaccinated fish and there was no difference in serum IL-2 levels between exposed and unexposed fish administered either the injection GroEL vaccine or the injectable sham vaccine preparations. However, fish administered the oral GroEL vaccine and challenged with *E. piscicida* S11–285 had significantly higher serum IL-2 levels at 28 dpc than unexposed and vaccinated fish, sham vaccinated and challenged fish, and negative control sham vaccinated and unexposed fish.

## 4. Discussion

In this study, formalin-killed *E. piscicida* preparations derived from representative isolates of the six bacterial clades stimulated significant specific antibody responses in rainbow trout, channel catfish, and hybrid catfish against *E. piscicida* isolate S11–285. This finding demonstrates these genetically distinct isolates share conserved antigens capable of eliciting cross-reactive antibodies across clades. The observed cross-reactivity of the antibody response across clades underscores the potential for broad-spectrum vaccine strategies, suggesting that a single vaccine candidate could confer protection against multiple variants of this important fish pathogen.

Channel catfish and hybrid catfish had significantly higher levels of antibodies to *E. piscicida* than rainbow trout, but this finding did not correspond to improved whole blood bactericidal activity. In fact, only rainbow trout demonstrated consistently improved whole blood bactericidal activity following immunization whereas the whole blood bactericidal activity of immunized catfish was either the same as or worse than negative control fish. Possible explanations for the lack of bactericidal activity despite high *anti*-*E. piscicida* IgM levels in the immunized catfish include lack of neutralizing antibody production, host-species differences in cell-mediated immune response, and differences in complement activity. Neutralizing antibodies are typically defined as antibodies that bind to a pathogen or pathogen-derived toxin at specific epitopes, subsequently preventing pathogen entry into host cells [42]. Traditional vaccine development often relies on the stimulation of targeted antibody production against these neutralizing epitopes, many of which are virulence factors, in order to prevent infection and accelerate clearance of the pathogen. Unfortunately, not all antibodies produced by an organism are neutralizing and thus serum antibody levels may not correlate with the development of protective immunity, particularly for facultative intracellular pathogens like *E. piscicida* that can escape the humoral immune response [42–44]. Species-dependent differences in complement activity and/or cell-mediated immune response to immunization could also explain the disparity between the ELISA and whole blood bactericidal activity results. Variability in lytic ability and specificity of complement as well as differences in cell-mediated cytotoxicity and specificity between fish species have been previously demonstrated, which could contribute to differences in whole blood bactericidal activity between catfish and rainbow trout whole blood [45–47]. Further investigation into the cell-mediated immune responses and complement-mediated immune responses of trout and catfish to *E. piscicida* immunization is necessary to definitively determine the mechanism behind these differences. Species-specific differences in immune mechanisms could also contribute to the lack of significant vaccine protection observed in the Chinook salmon despite the selected protein antigens demonstrating significant immunogenicity via our immunoproteomic approach in the catfish and trout. Another consideration is the difference in immunization degree days between the catfish and trout in this experiment. In poikilotherms, environmental temperature plays a crucial role in adaptive immune responses, with the immune effects of vaccines in fish being time × temperature (degree-days) dependent [48,49]. All fish species were sampled at the same time points throughout this experiment, however as the catfish were reared at a higher temperature (25 ^◦^C ± 1) than the rainbow trout (18 ^◦^C ± 1) they experienced higher average immunization degree-days (dd) at the time of serum collection (1050^◦^-days for catfish; 756^◦^-days for trout). As higher water temperatures, and thus higher degree-days, are associated with faster development of adaptive immune responses to vaccines in fish, this difference in degree-days at the time of sampling could explain the relatively higher specific antibody response to *E. piscicida* observed in the catfish. This could also possibly explain the difference in whole blood bactericidal response as the catfish and trout could be in different phases of the immune response at the time of sampling and thus bactericidal activity could have waned in the catfish [50].

Development of effective protein vaccines for genetically diverse pathogens such as *E. piscicida* relies on careful selection of highly conserved, surface-exposed, immunogenic protein antigens in order to induce long-lasting cross-protective immunity [51,52]. The shotgun immunoproteomic workflow outlined in this study presents a high-throughput method for identification of immunogenic bacterial proteins to drive vaccine candidate selection. In this study, protein G coated magnetic beads were used to capture antibodies from formalin-killed *E. piscicida* immunized trout and catfish serum to enrich immunogenic *E. piscicida* proteins for downstream identification. Prior to capture of serum antibodies, the magnetic beads were incubated with monoclonal mouse anti-trout/catfish IgM antibodies due to the low binding affinity of the protein G coated beads with IgM, the primary circulating antibody in teleosts. All 36 unique proteins determined to be immunogenic by this approach demonstrated high amino acid sequence homology among the twelve *E. piscicida* isolates used to generate the formalin-killed bacterial preparations in this study. Inclusion of highly conserved epitopes in protein vaccines is important for generation of broad strain/serovar protection; thus, our finding suggests these identified immunogenic proteins provide intriguing candidates for development of a protein vaccine with universal protection against *E. piscicida* clades. Additionally, most of the proteins identified by this method were also predicted to be probable antigens using *in-silico* methods, further validating the efficacy of this shotgun immunoproteomic approach to selectively identify immunogenic proteins.

Although the composition of the rainbow trout and hybrid catfish immunoproteomes differed significantly, except for one shared protein (Small ribosomal subunit protein uS17), they were both comprised primarily of cytoplasmic proteins with housekeeping functions. One protein, glycine zipper 2TM domain-containing protein, was predicted to be localized to the outer membrane and had 100 % sequence homology among the twelve selected *E. piscicida* isolates. The outer membrane proteins of many gram-negative bacteria have been investigated as antigens for vaccine development due to their important role in bacterial pathogenicity and their high surface exposure facilitating host immune system detection [53,54]. Glycine zipper 2TM proteins have previously been identified via reverse vaccinology approaches as potential vaccine candidates against *Aeromonas hydrophila*, thus their identification as immunogenic outer membrane proteins in our current study makes them promising candidates for recombinant protein subunit vaccine development [52]. Another interesting immunogenic protein identified in our study is Coproporphyrinogen-III oxidase (hemN), which plays an important role in heme biosynthesis by catalyzing the conversion of coproporphyrinogen III to protoporphyrinogen IX [55]. As heme synthesis is critical for aerobic and anaerobic respiration, hemN is an attractive anti-virulence target for therapeutics and vaccine development [56].

Chaperonin GroEL was particularly interesting as a potential target for recombinant vaccine development. GroEL is a ubiquitous heat-shock protein that plays an essential role in protein homeostasis. GroEL is highly conserved across both prokaryotic and eukaryotic organisms in which it functions to form a nanocage structure, with its co-chaperone GroES, to encapsulate substrates in order to prevent aggregation of unfolded proteins and to facilitate proper protein folding [57]. Although traditionally believed to be localized to the cell cytoplasm due to its primary function and lack of a secretion signal sequence or other export motif, surface-associated GroEL has been characterized in many pathogenic bacteria, including *E. coli* [58], *Salmonella enterica* serovar Typhimurium [59], *Helicobacter pylori* [60], and *Streptococcus agalactiae* [61]. Liu et al. (2016) identified GroEL in the outer membrane profile and secretome of *E. tarda*, further suggesting that GroEL is likely to be surface exposed in *E. piscicida*. Regulation of GroEL expression is modulated not only by environmental temperature, but also by other stress response factors during infection and extracellular GroEL has been implicated in bacterial adherence to and invasion into host cells [58,62]. Immunization with recombinant GroEL has been previously demonstrated to induce protective immunity against *Francisella orientalis* and *S. agalactiae* in tilapia (*Oreochromis* spp.) [61,63]. Additionally, DNA vaccines encoding for GroEL have been experimentally shown to stimulate the humoral and cellular immune response against *Nocardia seriole* and *E. tarda* in hybrid snakehead (*Channa argus* ♀ × *Channa maculate* ♂) and flounder (*Paralichthys olivaceus),* respectively [62,64].

These factors suggested that GroEL would be a promising candidate for recombinant protein vaccine development against *E. piscicida.* Thus, it was surprising that in this experiment, immunization with the injectable GroEL protein vaccine preparation generated an anti-protective effect in the Chinook salmon against subsequent *E. piscicida* challenge. One possible explanation for this finding is that molecular mimicry led to the development of auto-reactive antibodies and subsequent increased post-challenge mortality. In this context, molecular mimicry refers to the phenomenon in which significant similarities, such as sequence homology, between foreign peptides and self-peptides stimulate activation of autoreactive T and/or B cells that are cross-reactive with both pathogen-derived peptides and host peptides in a susceptible individual [65,66]. Heat shock proteins (HSPs) like GroEL are highly conserved across both prokaryotes and eukaryotes and sequence similarity between bacterial and host HSPs could lead to production of auto-reactive antibodies. There is evidence of cross-reactivity between antibodies generated against microbial heat shock proteins (HSPs) and eukaryotic 60-kDa HSPs contributing to the development of different autoimmune diseases in humans [67,68]. A similar phenomenon has been demonstrated in Atlantic salmon (*Salmo salar*) where vaccinated farm fish and experimentally vaccinated fish generated autoantibodies reactive against salmon cells as well as had higher rates of immune complex glomerulonephritis and arthritis [69–71]. When we compared the protein sequence of the *E. piscicida* GroEL protein sequence against the Chinook salmon proteome using BLASTP 2.16.0+, we found the bacterial GroEL had moderate (~52 %) sequence homology with the Chinook salmon Hsp60 protein (Supplemental Table 4). Western blot analysis did show a specific antibody response in the GroEL vaccinated salmon against Chinook salmon Hsp60, however it did reveal light reactivity of vaccinated salmon serum against a protein of ~40 kDa (Supplemental Fig. 1). It must be noted that protein conservation alone is not the sole etiology in the development of autoimmunity as bacteria and vertebrates share a great number of homologous proteins [65,72]. It is largely believed that additional host factors, such as genetic susceptibility, and external factors are necessary to upset the balance between autoimmunity and self-tolerance. Thus, although the exact cause of the anti-protective effect following GroEL vaccination observed in this experiment is still unclear, consideration of the potential for molecular mimicry is still warranted in future protein vaccine studies.

In contrast to the GroEL vaccine trial, the injectable COPIII and GlyZip vaccine formulations induced a small, but non-significant increase in post-challenge survival in this study despite inducing strong and specific *anti*-*E. piscicida* and anti-recombinant protein IgM responses in the vaccinated fish. These results are similar to reports in other studies that have shown a lack of significant vaccine protection despite high total IgM titers, demonstrating that total IgM levels alone are not necessarily indicative of vaccine efficacy [73–79]. As previously discussed, total IgM levels do not necessarily reflect functional IgM activity as not all immunoglobulins generated are neutralizing antibodies [42–44]. Going forward, assays evaluating the neutralizing capacity and effector functions, such as induction of phagocytosis or stimulation of cytokine release, of the antibodies generated by vaccinated fish may provide a clearer insight into the true functionality of the humoral response to immunization rather than simply measuring the quantity of antibodies produced [80,81]. Additionally, other immune mechanisms such as complement activity and cell-mediated immunity may play crucial roles in the development of a robust and effective immune response against the pathogen [18,44,45,47,82,83] leading to poor correlation between specific antibody titers and post-vaccination protective immunity. One possible explanation for the vaccine failure in this experiment is the protein vaccines did not stimulate a robust cell-mediated immune response which is necessary for effective clearance of a facultative intracellular pathogen such as *E. piscicida.* We did not find any significant differences in serum IL-2 levels between vaccinated and unvaccinated fish at 28 dpc. IL-2 plays a key role in teleost cell-mediated immunity through regulation of T helper 1 and T helper 2 cytokine expression [84]. Thus, this finding could suggest a lack of strong cell-mediated immune response from vaccination but could also be associated with the late timing of sampling (28 days post-challenge). Further investigation into differential regulation of factors associated with cell-mediated immunity following bacterial challenge is warranted to better elucidate the protective ability of the protein vaccines. Conversely, however, the slight, albeit non-significant, increase in survival could suggest that further optimization of dosing, booster, and/or delivery protocols may improve vaccine efficacy. Only one protein dose and a single booster was used for all vaccine formulations in this study, thus increased concentrations of purified protein and/or several boosters could potentially improve immune stimulation. Alternatively, a multi-epitope vaccine containing multiple immunogenic proteins could be considered to increase immunogenicity, stimulate multiple different immune pathways, and potentially increase protection across the genetically diverse *E. piscicida* strains [85,86]. The use of modern alternative delivery platforms, outside of the classical method of antigen suspension in adjuvant, could also be explored to improve antigen delivery and immune stimulation. Additionally, more targeted focus on surface-exposed antigens more readily detected by the host immune system could improve the success of vaccine candidate selection. The COPIII protein used in this experiment was determined to be highly immunogenic based on shotgun immunoproteomics and ELISA analyses with cell lysates. However, its localization to the cytoplasm could explain why high *in vitro* immunogenicity did not translate into significant protective effect *in vivo* as antibodies generated against these proteins may be ineffective in recognizing the protein, due to lack of surface exposure, or neutralizing live, intact bacteria. In this study, antisera used for shotgun immunoproteomics was generated via immunization of fish with formalin-killed bacterial preparations. Immunization with formalin-inactivated pathogens can lead to direction of antibodies against nonprotective epitopes due to alteration of functional epitopes necessary for protection [87]. Infection of fish with a live, virulent bacterial strain and harvesting of antisera from surviving fish may allow for more effective collection of neutralizing antibodies that are specific for protective bacterial antigens and preserve *in-vivo* antigen conformation and expression, thus improving identification of protective antigens. Additionally, the small sample size in this study (n = 24 fish/treatment) could have contributed to the non-significant results from the COPIII and GlyZip vaccine trial due to Type II error. In future studies, larger sample sizes are necessary to sufficiently detect smaller changes in protection rate following vaccination.

All injectable vaccinated fish, regardless of challenge status, demonstrated a strong specific antibody response against the immunizing protein, confirming the selected proteins were effective at stimulating a humoral response in the fish. A similar trend was not observed for the oral vaccine formulations, possibly due to degradation of the protein in the gastrointestinal tract and/or inadequate uptake by gastrointestinal lymphoid cells leading to failure of vaccination. Alternative methods to improve oral antigen delivery and antigen stability in the gastrointestinal tract could be explored to improve the efficacy of oral immunization with protein vaccine candidates. Additionally, sham vaccinated fish challenged with *E. piscicida* S11–285 also displayed a strong *anti*-*GroEL* antibody response compared to negative control fish, suggesting the salmon immune system was detecting and responding to the native GroEL protein of the bacteria. Interestingly, unchallenged fish immunized with the injectable GlyZip vaccine formulation demonstrated an increased *anti*-*E. piscicida* antibody response compared to both negative control fish and challenged sham vaccinated fish. This suggests the GlyZip protein was effective at stimulating an *anti*-*E. piscicida* humoral response regardless of challenge status. However, the development of a robust post-vaccination specific antibody response did not correspond to protective immunity in this study. Previous studies have found evidence that cell-mediated immunity may play a dominant role in the protection against *E. piscicida* while antibodies may provide only weak defense against the pathogen, possibly due to the ability of *E. piscicida* to invade host cells and replicate intracellularly [88,89]. This could explain the lack of significant protection observed in this study despite high specific antibody levels.

Although this study did not yield an effective protein vaccine candidate, it did demonstrate the utility of the described shotgun immunoproteomic approach for identifying immunogenic *E. piscicida* proteins and revealed critical differences in bacterial survival within the blood of salmonid vs. catfish hosts. The shotgun immunoproteomic approach described here vastly accelerates immunogenic protein identification over more laborious and time-consuming classical techniques, such as display technologies and Western blot, as well as bypasses the need for secondary protein immunogenicity validation required by computational methodologies. These findings lay an important foundation for future investigations into the host-pathogen dynamics of *E. piscicida* in these two economically significant aquaculture species, as well as provides an alternative method for high-throughput screening of the pathogen proteome for candidate antigens that can be immediately tested *in-vivo*. Moving forward, efforts should focus on targeting surface-exposed antigens more likely to be recognized *in vivo*, along with exploring alternative delivery strategies to enhance antigen uptake and stimulate a more robust and protective immune response.

## Supplementary Material

Multimedia component 2

Multimedia component 1

Multimedia component 3

Multimedia component 4

Multimedia component 5

## Figures and Tables

**Table 1:** List of *Edwardsiella piscicida* isolates and doses used in trout and catfish immunization.

Isolate	Clade	Source	OD	CFU/mL	Vaccine Group	References	NCBI Accession

6-30 IP D16-004	Clade I	Largemouth bass (*Micropterus salmoides*)	0.151	3.8 × 10^8^	V1A	Nguyen et al. (2021)	CP150945-CP150946
34-68^a^	Clade I	Atlantic salmon (*Salmo salar*)	0.158	1.3 × 10^8^	V1B	Nguyen et al. (2021)	CP150944
S11-233^a^	Clade II	Channel catfish (*Ictalurus punctatus*)	0.157	1.8 × 10^8^	V2A	Griffin et al. (2014)	CP150940
A15-1679	Clade II	Blotched fantail stingray (*Taeniura meyeni*)	0.163	6.0 × 10^8^	V2B	Camus et al. (2016)	CP150939
32-97	Clade III	Atlantic salmon (*S. salar*)	0.157	1.2 × 10^8^	V3A	Nguyen et al. (2021)	CP150938
35-70^a^	Clade III	Atlantic salmon (*S. salar*)	0.166	2.8 × 10^8^	V3B	Nguyen et al. (2021)	CP150937
S11-285	Clade IV	Channel catfish (*I. punctatus*)	0.153	6.7 × 10^8^	V4A	Griffin et al. (2014)	CP150934-CP150935
S12-281^a^	Clade IV	Channel catfish (*I. punctatus*)	0.154	1.5 × 10^8^	V4B	Griffin et al. (2019)	CP150931-CP150932
C1490	Clade V	Largemouth bass (*M. salmoides*)	0.154	1.5 × 10^8^	V5A	Fogelson et al. (2016)	CP150927-CP150928
LADL99-462^a^	Clade V	Channel catfish (*I. punctatus*)	0.150	6.2 × 10^8^	V5B	Griffin et al. (2014)	CP150925-CP150926
S16-278	Clade VI	Hybrid catfish (♀ *I. punctatus* × ♂ *I. furcatus*)	0.165	1.5 × 10^8^	V6A	Griffin et al. (2019)	CP150923-CP150924
MA97-004^a^	Clade VI	Tilapia (*Tilapia sp.*)	0.160	5.2 × 10^8^	V6B	Griffin et al. (2014)	CP150922

a Isolates were used for whole blood survival assays.

**Table 2:** Immunogenic *E. piscicida* proteins identified by shotgun immunoproteomics with LC/MS-MS. Differentially abundant proteins were determined to be immunogenic if there was a log2 ratio ≤ — 1 and *p*-value <0.05 between the vaccinated serum group and the negative control serum group (NC/Vaccinated).

Protein	Gene	Uniprot ID	Fish Species	GO Function	Predicted Localization

Chaparonin GroEL	*groL*	A0A411H1J6	Rainbow trout	Protein folding	Cytoplasmic
Peptidyl-prolyl cis-trans isomerase	EVK84_11485	A0A411H4M5	Rainbow trout	Protein folding Protein peptidyl-prolyl isomerization	Cytoplasmic
Universal stress protein	EVK84_01485	A0A411GZA8	Rainbow trout	No annotation available	Cytoplasmic
3-hydroxyacyl-[acyl-carrier-protein] dehydratase FabZ	*fabZ*	A0A411H0B7	Rainbow trout	Fatty acid biosynthetic process	Cytoplasmic
Translation initiation factor IF-3	*infC*	A0A411H6Y4	Rainbow trout	Translational initiation	Cytoplasmic
**Large ribosomal subunit protein bL32**	*rpmF*	A0A034SWU5	Rainbow trout	Translation	Cytoplasmic
**Large ribosomal subunit protein uL14**	*rplN*	A0A034SXP1	Rainbow trout	Translation rRNA binding	Cytoplasmic
**Large ribosomal subunit protein bL17**	*rplQ*	A0A411H351	Rainbow trout	Translation	Cytoplasmic
**Large ribosomal subunit protein uL22**	*rplV*	A0A034TB70	Rainbow trout	Translation rRNA binding	Cytoplasmic
Large ribosomal subunit protein bL34	*rpmH*	A0A411H281	Rainbow trout	Translation	Cytoplasmic
**Small ribosomal subunit protein uS5**	*rpsE*	A0A411H332	Rainbow trout	Translation rRNA binding	Cytoplasmic
**Small ribosomal subunit protein uS15**	*rpsO*	A0A034T1S1	Rainbow trout	Translation rRNA binding	Cytoplasmic
**Small ribosomal subunit protein uS17**	*rpsQ*	A0A034T846	Rainbow trout Hybrid catfish	Translation rRNA binding	Cytoplasmic
**Large ribosomal subunit protein uL30**	*rpmD*	A0A034SVG0	Hybrid catfish	Translation	Cytoplasmic
**Large ribosomal subunit protein bL33**	*rpmG*	A0A034T2A4	Hybrid catfish	Translation	Cytoplasmic
**Large ribosomal subunit protein bL27**	*rpmA*	A0A034T2R0	Hybrid catfish	Translation	Cytoplasmic
**Large ribosomal subunit protein uL29**	*rpmC*	A0A034T9U2	Hybrid catfish	Translation	Cytoplasmic
**Large ribosomal subunit protein uL24**	*rplX*	A0A411H335	Hybrid catfish	Translation	Cytoplasmic
DNA-binding protein HU-beta	EVK84_02390; *hupB*	A0A411GZR0	Hybrid catfish	DNA binding	Cytoplasmic
ATP-dependent Clp protease proteolytic subunit	*clpP*	A0A411GZW2	Hybrid catfish	ATP-dependent peptidase activity	Cytoplasmic
3,4-dihydroxy-2-butanone 4-phosphate synthase	*ribB*	A0A411H1B2	Hybrid catfish	Magnesium ion binding	Cytoplasmic
Bifunctional purine biosynthesis protein PurH	*purH*	A0A411H1V0	Hybrid catfish	’de novo’ IMP biosynthetic process	Cytoplasmic
NAD(P)H-flavin reductase	EVK84_06635	A0A411H1X0	Hybrid catfish	riboflavin reductase (NAD(P)H) activity	Cytoplasmic
Coproporphyrinogen-III oxidase	*hemN*	A0A411H2C0	Hybrid catfish	Protoporphyrinogen IX biosynthetic process; metal ion binding; coproporphyrinogen oxidase activity	Cytoplasmic
Aspartate-semialdehyde dehydrogenase	EVK84_13015; L1P05_RS11525	A0A411H535	Hybrid catfish	NAD binding; protein dimerization activity	Cytoplasmic
**DUF883 domain-containing protein**	EVK84_05270; L1P05_RS02125	A0A034SZJ7	Hybrid catfish	Ribosome binding	Cytoplasmic membrane
Sec translocon accessory complex subunit YajC	*yajC*	A0A411GZQ9	Hybrid catfish	No annotation available	Cytoplasmic membrane
Phosphatidylserine decarboxylase proenzyme	*psd*	A0A411H1G2	Hybrid catfish	Phosphatidylethanolamine biosynthetic process	Cytoplasmic membrane
(Fe-S)-binding protein	EVK84_05785; L1P05_RS01630	A0A411H1H8	Hybrid catfish	No annotation available	Cytoplasmic membrane
YgiW/YdeI family stress tolerance OB fold protein	EVK84_05170; L1P05_RS02225	A0A411H7T7	Hybrid catfish	No annotation available	Non-cytoplasmic
Glycine zipper 2TM domain- containing protein	EVK84_16935; L1P05_RS08220	A0A411H781	Hybrid catfish	No annotation available	Outer membrane
Superoxide dismutase	*sodB*	A0A411H779	Rainbow trout	Metal ion binding, superoxide dismutase activity	Periplasmic
UPF0757 protein YmgG	*ymgG*	A0A411GZ36	Hybrid catfish	No annotation available	Unknown
Glycerol dehydrogenase	EVK84_02840; L1P05_RS04435	A0A411GZZ6	Hybrid catfish	Metal ion binding; oxidoreductase activity	Unknown
Biotin carboxyl carrier protein of acetyl-CoA carboxylase	*accB*	A0A411H391	Hybrid catfish	Fatty acid biosynthetic process	Unknown
Negative modulator of initiation of replication	*seqA*	A0A411H4K0	Hybrid catfish	Negative regulation of DNA-templated DNA replication inhibition	Unknown

**Table 3:** Summary of vaccine trial design and fish survival.

Group	Primary Vaccination (Day 0)	Booster Vaccination (Day 28)	Challenge Groups (Day 42, 0.1 mL/fish)	CPM (%)	RPS (%)

Sham Vaccinated (Control)	PBS + ISA (IC)	PBS + ISA (IC)	PBS	0	-
			10^6^ CFU/fish Ep S11-285	22.2^a^, 60^b^	-
	PBS + GR1 (PO)	PBS + GR1 (PO)	PBS	0	-
			10^6^ CFU/fish Ep S11-285	21.7^a^, 38.2^b^	-
Vaccinated	GroEL + ISA (IC)	GroEL + ISA (IC)	PBS	0	-
			10^6^ CFU/fish Ep S11-285	63.1	−184
	GroEL + GR1 (PO)	GroEL + GR1 (PO)	PBS	0	-
			10^6^ CFU/fish Ep S11-285	38.1	−76
	COPIII + ISA (IC)	COPIII + ISA (IC)	PBS	0	-
			10^6^ CFU/fish Ep S11-285	56.5	6
	COPIII + GR1 (PO)	COPIII + GR1 (PO)	PBS	0	-
			10^6^ CFU/fish Ep S11-285	44	−15
	GlyZip + ISA (IC)	GlyZip + ISA (IC)	PBS	0	-
			10^6^ CFU/fish Ep S11-285	54.2	9.7
	GlyZip + GR1 (PO)	GlyZip + GR1 (PO)	PBS	0	-
			10^6^ CFU/fish Ep S11-285	33.3	13

ISA, Montanide ISA 763 A adjuvant; GR1, Montanide GR1 adjuvant; IC, intra-coelomic administration; PO, oral administration; GroEL, purified Chaparonin GroEL protein; COPIII, purified coproporphyrinogen-III oxidase protein; GlyZip, purified glycine 2TM zipper domain-containing protein; Ep, *Edwardsiella piscicida*; CPM, cumulative percent mortality; RPS, relative percent survival; -, not applicable.

a: CPM from GroEL vaccine trial;

b: CPM from COPIII and GlyZip vaccine trial.

## Data Availability

The data presented in this study are available on request.

## Appendix A. Supplementary data

Supplementary data to this article can be found online at https://doi.org/10.1016/j.fsi.2025.110992.
