# Supplementary material for: Use of shotgun immunoproteomics for the development of protein vaccines against Edwardsiella piscicida
Source: Fish Shellfish Immunol. Author manuscript; Available in PMC 2026 Sep 4. (PMC13542899; doi:10.1016/j.fsi.2025.110992)
Supplement: Supplementary file 1 [file NIHMS2205224-supplement-Multimedia_component_3.docx]

**Supplemental Table 3.** cDNA sequences of *E. piscicida* (A) Chaparonin GroEL, (B) coproporphyrinogen-III oxidase, and (C) Glycine zipper 2TM domain-containing protein genes submitted to Biomatik for protein expression and purification.

A. GroEL DNA sequence

1 atggcagcta aagacgtaaa atttggtaat gacgcccgcg taaaaatgct gcgcggcgtg

61 aacgttctgg cagacgcagt gaaagtgacc ctgggtccga aaggccgtaa tgtggtcctg

121 gataaatcct tcgggtctcc gaccatcacc aaagacggcg tgtccgttgc gcgtgaaatc

181 gaactggaag acaagttcga gaacatgggc gcgcagatgg tgaaagaagt cgcctccaag

241 gcgaatgacg ccgccggtga cggcaccacc accgcaaccg tactggcaca ggctatcgtt

301 aacgaaggtc tgaaagccgt ggctgcgggt atgaacccga tggatctgaa gcgcggcatc

361 gacaaagccg tgaccgccgc cgttgaagag ctgaaggcgc tgtctgtacc gtgctctgac

421 tctaaagcca ttgcgcaggt agggaccatc tccgccaacg ccgacgacac cgtgggccag

481 ctgatcgctg aagccatgga aaaagtgggt aaagaaggcg ttatcaccgt tgaagaaggc

541 accggtctgc aggacgagct ggacgtggtt gaaggtatgc agttcgatcg cggctacctg

601 tctccgtact tcatcaacaa gccggaaacc ggcgccgttg agctggaaag cccgttcatt

661 ctgctggctg acaagaagat ctccaacatc cgcgaaatgc tgccggtgct ggaagccgtt

721 gctaaagccg gtaagccgct gctgatcatc gccgaagacg ttgaaggcga agcgctggcg

781 accctggtgg tcaacaacat gcgcggtatc gtgaaagtgg ctgcggtgaa agcgccgggc

841 ttcggcgatc gtcgtaaggc gatgctgcag gatatcgcta ccctgaccgc cggtaccgtt

901 atctctgaag agatcggtat ggagttggaa aaagcgacgc tggaagatct gggccaggct

961 aaacgcgtgg tcatcaacaa agacaccacc attatcatcg atggcgtggg cgaagagtct

1021 gccattcagg gtcgtgttac tcagatccgt cagcagatcg aagaagcgac ctctgactac

1081 gatcgtgaaa aactgcagga gcgcgtggct aaactggcgg gcggcgtagc cgttatcaag

1141 gttggcgcgg caactgaagt tgaaatgaaa gagaagaaag cacgcgtaga agacgccctg

1201 cacgcaaccc gtgctgcggt ggaagaaggc gtggtggccg gcggcggcgt ggcgctggtt

1261 cgcgtagcgg ccaagctggt tgatctgaaa ggcatcaacg aagagcagaa cgtcggtatc

1321 aaggttgcgc tgcgcgcgat ggaagcgccg ctgcgtcaga tcgtggccaa cgccggtgaa

1381 gagccgtcag tggtcaccaa caacgtgaag gccggcgaag gtaactacgg ctataacgcg

1441 gcgaccgaag agtacggcga catgatcgat atgggtatcc tggatccgac caaggtaacc

1501 cgctctgcgc tgcagtacgc ttcctctgtt gccggtctga tgatcaccac cgagtgcatg

1561 gtgaccgatc tgccgaagga agagaaagct gacctgggtg ctgccggtat gggtggcatg

1621 ggcggtatgg gcggcatgat gtaa

B. Coproporphyrinogen-III_oxidase DNA sequence

1 atgtcagaac agactatcgt ctgggatttg gccctgatcc agaagtataa ctattccggg

61 ccgcgctata cctcgtaccc caccgcgcta gagtttaacg aaggctttga tgaggcggcc

121 tttctgcgtg ccgtagcccg ctaccccgat cgggcgcttt ccctttacgt gcatatcccg

181 ttctgccata agctgtgcta cttctgcggc tgcaataaga tagtgacccg ccagtctcac

241 aaggccgacg agtatttgga taagctggcg ctggagatcg ccgctcgcgc gccgctgttt

301 gccgggcgga cggtgagcca gctgcattgg ggcggcggta cgccgacctt tctcaataag

361 gcgcagatta gccgcctcat gacgctgctg cgtcgccatt ttacctttgc gcctcaggcg

421 gagatttcga tagagatcga tccgcgggag attgcgttgg atgtgttgga tcacctgcgc

481 caagaagggt ttaaccgcct gagcatgggg gttcaggact tcaataagac cgtgcagacg

541 ttggtcaacc gcgagcagga tgaggagttt atcttttccc tgctgcgcca tgcgcgggaa

601 atcggcttta cctctaccaa tatcgatctg atttatggtc tgcctcgcca gacgccggag

661 agcttcgatt ttacgctgca aaaggtgatc gagctgaatc ccgatcgact cagcattttt

721 aactatgcac acatgcctaa gctgtttgcc gctcagcgta agatcaaaga tgccgatctg

781 cccagcgcag accataagct ggccattcta cagcagagca tcgcgacgct gaccggcgtg

841 ggataccagt ttatcgggat ggatcacttt gcccaccgtg acgatgagct ggcggtggcc

901 cagcgcgccg gtaagctgca tcgcaacttt caggggtata ccacgcaggg cgacagcgat

961 ttgctggggc tgggcgtatc ggcgattagc atgatcgggg atagctatgc ccaaaaccgc

1021 aaggtgctga aggagtacta tgccgcggtt gaggcgcagg gcaacgcgct gtggcgcggg

1081 ctggctatga gccgagatga ctgtattcgc cgcgatgtga ttaaggggct tatctgtaat

1141 ttccgcctgg atttcgcgcc gatagaacag cagtacggca ttcgctttgt ggattatttc

1201 atcgaagatc tgctgctgct gcgtccgctg gcgcaggatg ggctggtcga gctgggtgag

1261 acggcgatta acgttacgcc gcgcggccgt ctgctgatcc ggaatatctg catgtgcttt

1321 gatgtgtatc tgcgtcatca ggcccgaatg cagcagtttt cgcgcgttat c

C. Glycine zipper 2TM domain-containing protein DNA sequence

1 atgatgaagc gtcttattat tgtcgctatt gccacggcaa ccctggcggg ctgtgccaat

61 accagcaccc tgtccggcga cgtgtacagc gccaatcagg ccaagcaggt acagcaggtg

121 acatatggca ccatcgtctc catggagccg gtacagattc aagccggtag cgaggcgaac

181 atgatcggca ccatcggcgg cgccgtcatc ggggggctgc tgggtaacac catcggcggc

241 ggcaccggac gcagcctggc gaccgccgca ggcgccgtcg cgggcggcgt ggccggcaac

301 agcattgagg gcgcggtcaa ccgtaccaac ggcgtccagc tggtgatccg tctcgacagc

361 ggtaagacca tcgccgtggt acagaaaaac gacaagcagg ctttccgggt taatcagcgc

421 gtaatgctgc tgagcaacgg cagcaccgtt aacgtctcgc cgatg
