# Supplementary material for: Use of shotgun immunoproteomics for the development of protein vaccines against Edwardsiella piscicida
Source: Fish Shellfish Immunol. Author manuscript; Available in PMC 2026 Sep 4. (PMC13542899; doi:10.1016/j.fsi.2025.110992)
Supplement: Supplementary file 2 [file NIHMS2205224-supplement-Multimedia_component_5.docx]

**Supplemental Figure 1.** SDS-PAGE and western blot analysis of purified Chinook salmon head kidney protein. (A) SDS-PAGE of total Chinook salmon head kidney proteins (Lanes 2-3). Molecular mass markers are indicated to the left (Lane 1). (B) Western blot analysis of Chinook salmon proteins using serum from salmon vaccinated with the injectable GroEL vaccine preparation (C3-1 to C3-5; lanes 7-11) and serum from salmon administered the injectable sham vaccine (C1-1 to C1-5; lanes 1-5). Molecular mass markers (kDa) are indicated in lanes 6 and 12.

**B**

**A**

**1	2	3	4	5	6	7	8	9	10	11 12**

**3**

**2**

**1**
